# Emerging antibody-drug conjugate (ADC) therapies: perspectives from the global patent landscape

**DOI:** 10.3389/fphar.2026.1853273

**Published:** 2026-10-22

**Authors:** Tong Wu, Baijun Liu, Jiayu Jin, Yajie Zhou, Hongmei Yuan

**Affiliations:** 1 School of Business Administration, Shenyang Pharmaceutical University, Shenyang, China; 2 Key Laboratory of Environmental Medicine and Engineering of Ministry of Education, Department of Nutrition and Food Hygiene, School of Public Health, Southeast University, Nanjing, China

**Keywords:** antibody-drug conjugate, BERTopic, high-impact patent, knowledge flow, patent analysis, topic citation network

## Abstract

**Background:**

Antibody-drug conjugates (ADCs) represent one of the most significant innovations in biopharmaceutical research, integrating antibody target specificity with the cytotoxic potency of small-molecule payloads. Despite rapid progress, the technological structure and knowledge dynamics underlying ADC innovation remain insufficiently characterized. This study aimed to map the ADC technological landscape through patent analysis of 6,620 patent families published before 30 June 2025.

**Method:**

BERTopic was applied to extract global topics, and characterize the ADC knowledge structure. A directed patent citation network was constructed at the patent family level, and patent citations were aggregated by dominant topic to establish a topic citation network. Degree-based and PageRank metrics were used to identify key topics and evaluate their functional roles in knowledge flow.

**Results:**

The analysis categorized 47 technological topics into nine clusters covering Target Selection, Biological Mechanisms, Antibody Structure, Therapeutic Indications, Conjugation Chemistry, Linker Chemistry, Payload Modality, Delivery Systems, Formulations and Stability. Ten key topics were identified as hubs within the topic citation network, comprising five target topics: B-cell surface antigens, B7 family antigens, Mesothelin, AXL, and HER family receptors; two payload topics: PBD payloads and Camptothecin-derived payloads; two mechanism topics: Interleukin immunocytokine and Immune checkpoint modulation, and one delivery topic: Nanoparticle delivery. Topic role analysis indicated distinct patterns among absorption-oriented, diffusion-oriented, and bridging topics in shaping knowledge absorption, dissemination, and trans-topic reorganization.

**Conclusion:**

This study provides an evidence-based view of the ADC patent landscape and offers strategic insights for target prioritization, payload design, and R&D planning. It also demonstrates the applicability of integrated patent landscape analysis to other emerging technology fields.

## 1 Introduction

Antibody-drug conjugates (ADCs) are targeted therapeutics that covalently couple antibodies to potent payloads through chemical linkers (Fu et al., 2022). The antibody component selectively binds to antigens on cancer cell surfaces, whereas the payload, typically a small molecule cytotoxic agent, induces cell death (He et al., 2024). This architecture is designed to concentrate cytotoxic activity preferentially in antigen-expressing cells and limit systemic exposure. However, it creates a multi-parameter design space (Lin et al., 2025). Relevant determinants are antigen biology (expression heterogeneity, internalization, and shedding), antibody format and engineering modifications, linker stability and release mechanisms, drug-to-antibody ratio (DAR), conjugation homogeneity, and payload physicochemical properties (Colombo et al., 2024).

Clinical translation of ADCs has accelerated in recent years. By 2025, approximately a dozen ADCs had received regulatory approval, and over 200 candidates were undergoing clinical development for hematologic malignancies and solid tumors (Wang et al., 2025). Landmark trials, including those of trastuzumab deruxtecan (T-DXd) for HER2-low metastatic breast cancer and sacituzumab govitecan (SG) for metastatic triple-negative breast cancer (mTNBC), demonstrate that novel ADCs can expand eligible patients beyond conventional antigen selection (Bardia et al., 2021; Modi et al., 2025). Innovation in antibodies, linkers, and payloads has driven recent progress in ADC development. Conjugation chemistry has improved ADC homogeneity, stability, and performance. Key advances include site-specific conjugation for more precise control of conjugate heterogeneity, linker engineering that balances plasma stability with efficient intracellular or microenvironmental release, and payload diversification from microtubule inhibitors to topoisomerase I (Topo I) inhibitors and DNA-damaging agents. Collectively, these changes have enhanced efficacy and, in selected settings, may contribute to bystander effects and immunomodulatory activity (Li et al., 2025).

Nevertheless, ADC development remains vulnerable to failure in later stages. Biological resistance mechanisms, including target downregulation, altered intracellular trafficking, and efflux transporter activation, together with toxicity risks connected to target distribution and payload mechanism, continue to limit dose intensity and response durability (Loganzo et al., 2016). Challenges in manufacturing and formulation also constrain development, scale-up, and lifecycle management, particularly reproducible control of conjugation, aggregation reduction, and long-term stability (Nguyen et al., 2023).

Research on patent information demonstrates that patents provide a valuable complementary source for academic and industrial applications (Chen, 2003). Compared with scientific publications, patent documents frequently disclose chemical design principles, conjugation processes, and formulation strategies at earlier stages, providing an evidence base for technology exploration and innovation analysis (Trajtenberg, 1990). Although numerous patent reviews and research papers on ADCs have been published, systematic data-driven mining across the ADC field remains limited (Chia, 2022). Patent landscape analysis can organize dispersed patent information into coherent and traceable technological overviews, improving transparency in ADC technology research. Methodology guidelines from the World Intellectual Property Organization (WIPO) offer a standardized framework for such analysis (Trippe, 2015). Patent landscapes generated using WIPO methodology provide comparable and reproducible evidence, assisting stakeholders evaluate market dynamics and technological trends (Smith et al., 2018).

Previous ADC patent research examined the field from different perspectives. ADC was used as a case study to demonstrate the integration of various software tools in patent landscape, which improved the efficiency of ADC patent search and analysis (Masiakowski and Wang, 2013). Patent reviews have also addressed the protection of ADC components and complete conjugates, operational freedom considerations, and patents covering approved ADC products (Storz, 2015). Statistical analysis of global and Chinese patent applications described application trends, major applicants, therapeutic targets, and small molecule toxins (Chen et al., 2021). Recent research have adopted a broader scope. Scientific publications and patents were jointly analyzed to characterize research growth, geographic and organizational activity, main technological components, and emerging research directions (Sasso et al., 2023). ADC technologies were also reviewed together with market, clinical trials, and patent application information, providing an updated overview of technological development and major industrial participants (Choi et al., 2024). These studies established the technological and intellectual property context of ADC development, but mainly emphasized descriptive distribution, product protection, or overall research progress. This study extends this work by identifying technological domains across a global patent family corpus and connecting them through a topic-level citation network, thereby examining the knowledge structure within the ADC patent landscape.

This study conducts a reproducible analysis of the ADC technology patent landscape by integrating large-scale topic modeling with citation network analysis. Through merging technology topics with citation data to construct a topic citation network, the study examines inter-topic knowledge exchange and functional roles within the evolving ADC innovation ecosystem (Trajtenberg, 1990). The resulting technological landscape provides a basis for identifying frontier directions and strategic opportunities in ADC development.

## 2 Data and methods

A multilayer data-driven framework was established to explore the technological structure and knowledge flow of ADC technology from a patent perspective. As illustrated in Figure 1, the framework embedded semantic topic modeling within citation network analysis to map knowledge flow trajectories. It bridges unstructured information of patent data to structured evolution of technological knowledge.

**FIGURE 1:**
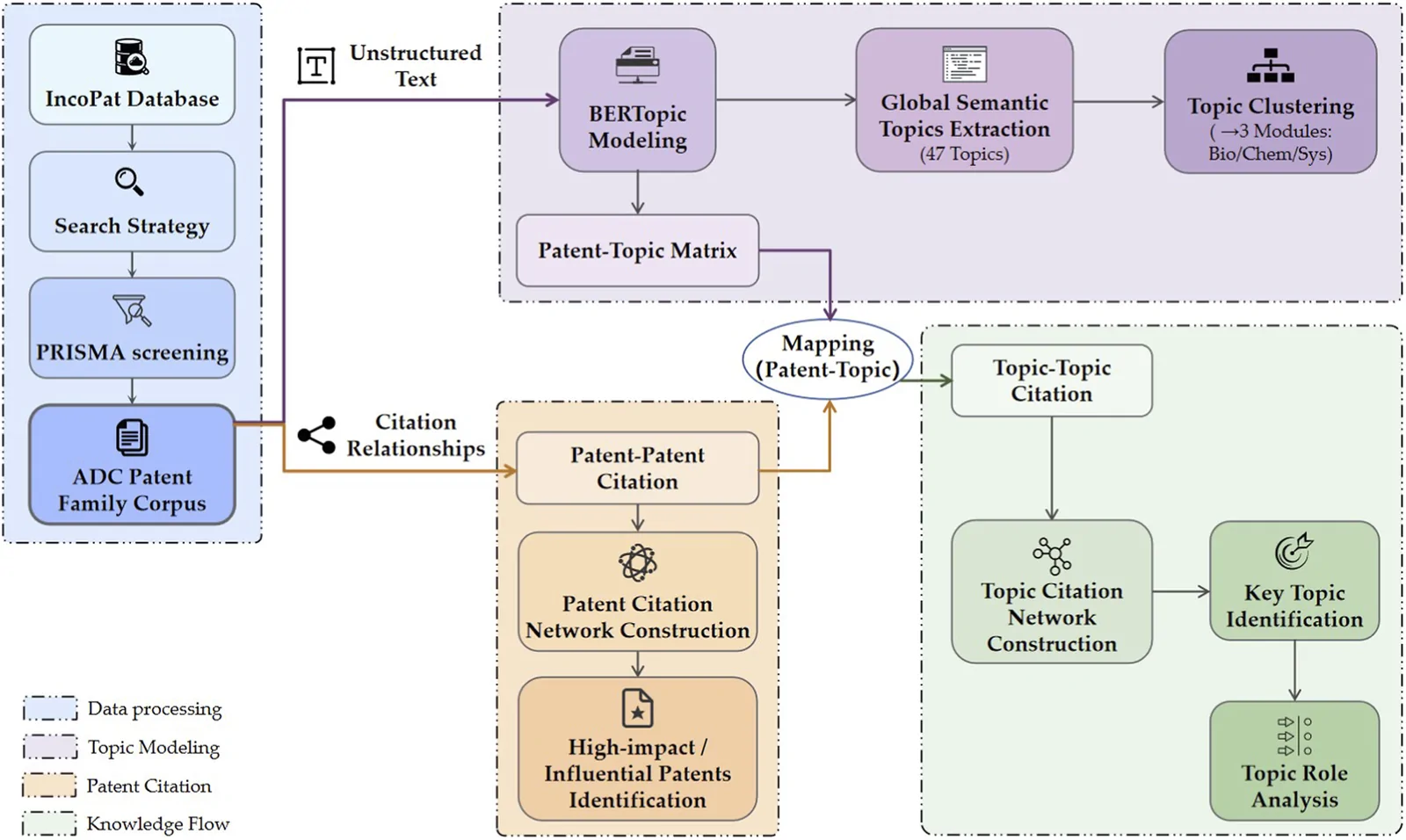
The research framework.

### 2.1 Data collection and preprocessing

Patent data were retrieved from the IncoPat database, which standardizes patent family structures based on the Derwent World Patent Index (DWPI), one of the most authoritative global repositories of enhanced patent information curated by Clarivate Analytics Patent retrieval focused on invention patents related to ADCs and broader conjugate technologies. The full search strategy was as follows: (TIAB = (“drug conjugate*” OR “immunoconjugate*” OR ((payload OR cytotoxic) AND (linker OR “site specific”)))) AND (AD = [19000101 TO 20250630]). The patent type encompasses both granted patents and invention applications, covering mature technologies and emerging innovations under research and development (R&D).

To standardize the dataset, records were organized into patent families based on the DWPI, with the earliest published document selected as the representative record for each family. This approach reduced duplicates arising from multiple filings for the same invention across jurisdictions, ensuring analysis units represented technological innovations rather than legal events. The search was performed on 16 July 2025, yielding an initial dataset of 28,286 individual patents, corresponding to 7,671 patent families. The dataset thus consisted of patent family level information, including patent numbers, titles, abstracts, claims, citation data, and other unstructured information.

A structured screening protocol based on PRISMA was applied to refine the dataset (Figure 2). This multistage filtering process prioritized semantic relevance over broad keyword matching. Exclusion criteria removed nonpharmaceutical conjugates, physical mixtures, and irrelevant industrial or agricultural applications. Only patents describing covalent conjugation or linker structures, such as sulfides, hydrazones, or cleavable linkers, were retained. Following screening, the final dataset comprised 6,620 highly relevant patent families.

**FIGURE 2:**
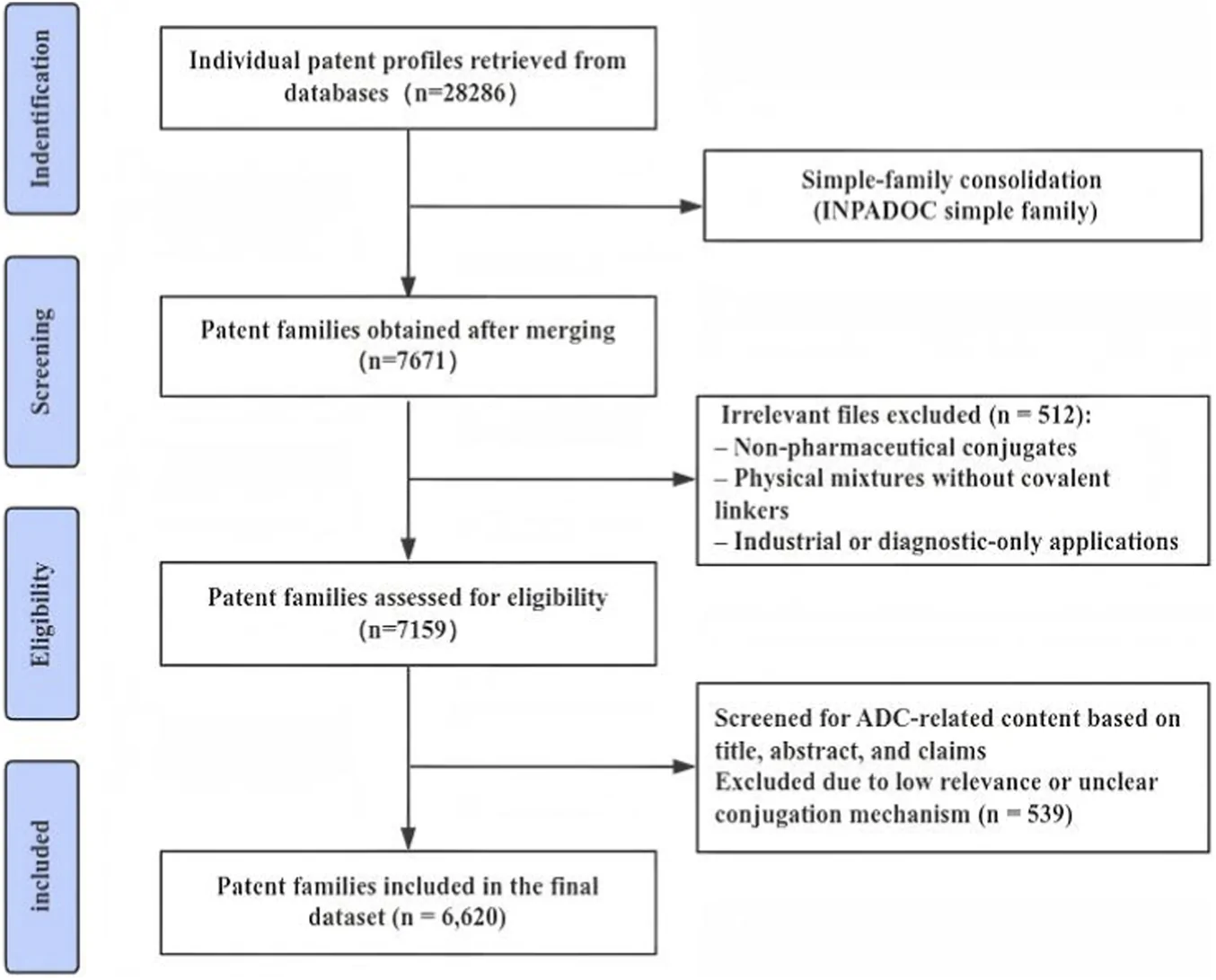
PRISMA flow diagram for ADC patent screening process.

### 2.2 Topic modeling with BERTopic

To uncover the latent thematic structure of the ADC patent landscape, BERTopic was employed. Transformer embeddings generated dense vector representations of text, overcoming the sparsity and limited contextual sensitivity of contextual bag-of-words models such as Latent Dirichlet Allocation (LDA) (Gan et al., 2023).

Patent titles and abstracts, which provide concise summaries of core technical content, were incorporated into the analytical corpus (Choi and Woo, 2022; Ghaffari et al., 2023). Before BERTopic modeling, text preprocessing included tokenization, stemming, and removal of stop words (and stop phrases). Generic English stop words and uninformative patent legal terms were filtered out, while domain-specific multiword expressions were retained to maintain semantic integrity in biomedical and chemical contexts (Hirave and Hanchate, 2015).

As illustrated in Figure 3, document embeddings were generated using the pretrained Sentence-BERT model all-MiniLM-L6-v2 to capture contextual information at the document level. To mitigate the curse of dimensionality, Uniform Manifold Approximation and Projection (UMAP) was applied to project high dimensional embeddings onto a lower dimensional manifold, preserving neighborhood relationships and global topological structure. Hierarchical Density-Based Spatial Clustering of Applications with Noise (HDBSCAN) was then applied to group semantically similar patent documents. This approach does not require a preset number of groups and is particularly advantageous for patent data. Finally, topic representations were derived using class-based term frequency-inverse document frequency (c-TF-IDF) weighting (Raman et al., 2024).

**FIGURE 3:**
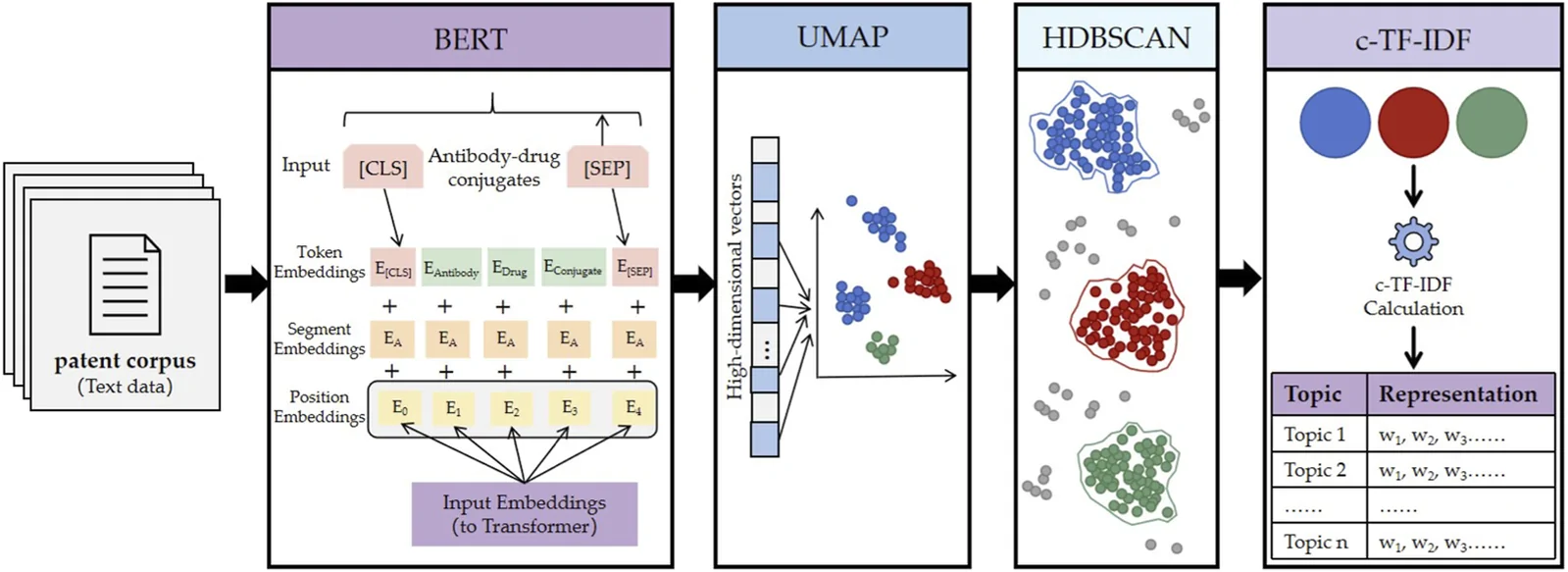
Process of BERTopic modeling.

BERTopic (version 0.16.4) was used for topic modeling. Patent texts were encoded with all-MiniLM-L6-v2 into 384-dimensional embeddings, using a maximum input length of 512 tokens, a batch size of 32, and mean pooling. The resulting embeddings were L2-normalized and reduced to 100 dimensions by principal component analysis, with a random seed of 42. UMAP was configured with n_neighbors = 10, n_components = 5, min_dist = 0.1, metric = “cosine”, and random_state = 42. HDBSCAN was applied with min_cluster_size = 15, min_samples = 5, Euclidean distance, a cluster selection epsilon of 0.05, and the leaf selection method. Topic representations were generated from unigrams and bigrams occurring in at least five documents and no more than 85% of the corpus, using the customized domain tokenizer described above. The c-TF-IDF calculation incorporated BM25 weighting and frequent-word reduction.

All eligible patent families were first incorporated into the BERTopic workflow. Iterative refinement involved consolidation of overlapping topics and remodeling of initially ambiguous or noise-classified records. Only patent families with stable dominant topic were retained for subsequent topic-level analyses.

### 2.3 Citation network and knowledge flow

To trace knowledge diffusion and technological evolution within the ADC innovation landscape, a multistage approach was constructed by integrating patent citation network analysis with topic citation analysis. The workflow first modeled citation relationships at the patent level and subsequently aggregated at the topic level to reveal knowledge flow. Network visualization and metric calculation were executed using Gephi software (v0.10.1) (Fruchterman and Reingold, 1991; Bastian et al., 2009).

#### 2.3.1 Construction of the patent citation network

Citation relationships among patents within a technology domain provide a structured perspective for examining technological relationships and development trends (Roach and Cohen, 2013). A directed citation network was constructed using patent-level citation, in which each patent family is both a citing and a cited entity (Waltman et al., 2010). To ensure topological robustness, multiple citation records between the same pair of families were aggregated into one weighted edge representing citation frequency (Chandra and Dong, 2020). Intra-family citations were excluded to eliminate bias arising from internal knowledge reinforcement (Xuan et al., 2024).

For a given patent family p , in-degree and out-degree were defined as the numbers of incoming and outgoing citation edges, capturing downstream knowledge dissemination and upstream knowledge absorption, respectively (Freeman, 1978). To jointly account for both dimensions, a cross-degree index was introduced to characterize the structural importance of each patent family node in the directed network. Let $d_{in}\left(p\right)$ and $d_{out}\left(p\right)$ denote the in-degree and out-degree, respectively. The cross-degree $d\left(p\right)$ is defined as:

$$d\left(p\right)={\left(d_{in}\left(p\right)+1\right)}^{\lambda }{\left(d_{out}\left(p\right)+1\right)}^{1-\lambda }-1$$

Where $\lambda \in \left[0,1\right]$ governs the relative weighting of knowledge dissemination versus absorption. Setting λ=0.5 gives equal weight to both dimensions. λ>0.5 emphasizes incoming citations (knowledge outflow), while λ<0.5 emphasizes outgoing citations (knowledge inflow). The cross-degree therefore provides a balanced indicator of the dual functions of a patent family in knowledge absorption and dissemination.

#### 2.3.2 Construction of the topic citation network

Building upon the patent citation network, semantic structures extracted from BERTopic were integrated to construct a topic citation network, extending the analysis from patent-level citations to topic-level knowledge flow (Ghaffari et al., 2023). Each patent family was assigned to its dominant topic, and citation relationships among families were aggregated to generate directed weighted edges among topics. Edge weights represent the total number of citation records between patents assigned to topic based citation relationships (Cheng and Zheng, 2025).

The construction process is illustrated in Figure 4, which maps patent-level citations to topic-level knowledge flows. When patents assigned to topic *A* cited those assigned to topic *B*, a directed edge was created from *A* to *B* in the topic citation network, representing the knowledge transfer from topic *B* as prior art to topic *A* as subsequent development.

**FIGURE 4:**
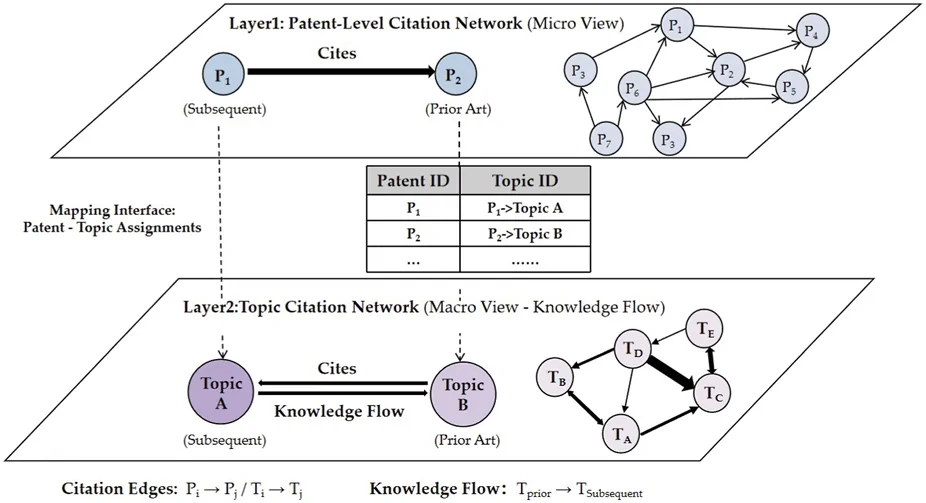
Construction process of topic citation network.

Within the directed weighted network $G_{T}=\left(V_{T},E_{T}\right)$ , each topic node possesses in-degree and out-degree measures that characterize its capacity for knowledge dissemination and absorption. Let $W_{ij}$ denote the aggregated citation weight from topic i to topic j . The weighted in-degree and weighted out-degree of topic t are given by

$$d_{in}\left(t\right)=\sum_{i}W_{it}$$

$$d_{out}\left(t\right)=\sum_{j}W_{tj}$$

Here, $d_{in}\left(t\right)$ captures the total citation weight received by topic t from all other topics, whereas $d_{out}\left(t\right)$ reflects the total citation weight from topic t to other topics. Consistent with the patent-level network, the cross-degree indicator was extended to the topic-level. The cross-degree of topic t is defined as:

$$d\left(t\right)={\left(d_{in}\left(t\right)+1\right)}^{\lambda }{\left(d_{out}\left(t\right)+1\right)}^{1-\lambda }-1$$

Where 0≤λ≤1 balances the contributions of in-degree and out-degree ( λ=0.5 assigns them equal weight). A higher $d\left(t\right)$ value indicates a topic with substantial citation inflows and outflows suggesting a bidirectional intermediary for knowledge dissemination and absorption within the network.

In addition to degree-based indicators, PageRank centrality was calculated for the topic citation network using the same weighted adjacency matrix $W_{ij}$ and a damping factor of 0.85. The PageRank score of a topic reflects its global citation prominence within the network, corresponding to the probability that a random walker following citation edges will arrive at that topic in the long run. Topics with high values in both PageRank and cross-degree were interpreted as key nodes that organize and diffuse technology knowledge at the system level.

#### 2.3.3 Topic roles assignment in knowledge flow

From the perspective of knowledge flow, each topic represents a knowledge unit that may absorb and disseminate technological knowledge to varying degrees. Within the citation framework used in this study, each directed edge represents a citation relationship from the citing entity to the cited entity. Knowledge flow was interpreted in the opposite direction to citations, transmitting the knowledge contained within the cited topic to the citing topic. The role of a topic was determined by its position in the overall knowledge flow.

Key topics were identified based on the topic citation network and assigned topic roles within the knowledge flow. A key topic was defined as a node with high values for both cross-degree and PageRank. The in-degree of a topic indicates the weight of other topics that cite this topic, capturing the extent to which its knowledge influences subsequent topics. Conversely, the out-degree represents the weight of its cites to other topics, reflecting the breadth of upstream knowledge sources. To characterize topic roles in knowledge flow, the diffusion rate was defined as:

$$r_{diff}\left(t\right)=\frac{d_{in}\left(t\right)}{d_{in}\left(t\right)+d_{out}\left(t\right)}$$

A higher $r_{diff}$ indicates a stronger tendency toward knowledge dissemination, whereas a lower $r_{diff}$ indicates greater reliance on knowledge absorption. Conceptually, topics roles were categorized as diffusion-oriented, absorption-oriented, and bridging. Bridging topics are identified as those with intermediate $r_{diff}$ values, which have simultaneously high in-degree and out-degree. Thresholds that distinguish these categories are empirically determined based on the distribution of $r_{diff}$ values, while retaining a reasonable number of key topics in each category.

## 3 Results

### 3.1 Topic modeling and technology identification

Patent titles and abstracts from 6,620 patent families were used as the primary textual sources for BERTopic modeling to capture core technical content while avoiding the legal complexity of claims. Following iterative refinement through overlapping topic merging and noise document backfilling, 6,553 patent families (99.0%) were identified 47 coherent global topics (Topic 0–46). The remaining 67 families (1.0%) were not included in the topic-level results because a stable dominant topic assignment could not be established.

Each topic represents a distinct technological domain characterized by high weight terms. Table 1 outlines the corresponding representative words and cluster assignment of 47 topics. According to their positions in the ADC development process, these topics were grouped into 9 technological clusters: Target Selection, Conjugation Chemistry, Payload Modality, Biological Mechanism, Delivery System, Therapeutic Indication, Antibody Architecture, Linker Chemistry, and Formulation and Stability. Their hierarchical structure is shown in Figure 5, where target and mechanism domains account for the largest share of the patent landscape. For interpretive clarity, the following subsections examine nine clusters along three higher level modules. The biological module includes Target Selection, Biological Mechanism, Antibody Architecture, and Therapeutic Indication. The chemical module includes Conjugation Chemistry, Linker Chemistry, and Payload Modality.The system module includes Delivery System and Formulation and Stability.

**TABLE 1:** ADC topics with corresponding technologies and clusters.

Technology cluster (%)	Topic ID	Topic label	Representative words (n = 10)
Target selection (38.8%)	1	Immunomodulatory targets	‘lag3’, ‘ligand-drug conjugate’, ‘ptk7’, ‘cd73’, ‘immune response’, ‘glp1’, ‘lymphoma’, ‘multiple myeloma’, ‘B cell lymphoma’, ‘non-hodgkin lymphoma'
	2	B-cell surface antigens	‘cd79b‘, ‘cd20’, ‘cd37’, ‘adjuvant’, ‘hematopoietic’, ‘internalization’, ‘cell-surface receptor’, ‘tumor-associated antigen’, ‘cd40’, ‘cdh6′
	4	B7 family antigens	‘b7h3’, ‘eribulin’, ‘b7h4’, ‘her2’, ‘her3’, ‘erbb2’, ‘albumin’, ‘non-small-cell lung’, ‘nsclc','calicheamicin'
	11	EGFR	‘egfr’, ‘epidermal growth’, ‘epidermal’, ‘erbb2’, ‘multispecific egfr’, ‘nsclc’, ‘lysine’, ‘cterminal’, ‘kabat’, ‘tumor resistant'
	13	HER family receptors	‘her2’, ‘her3’, ‘bcl2’, ‘breast cancer’, ‘her2-positive breast cancer’, ‘eribulin’, ‘trastuzumab’, ‘her2 cytotoxic’, ‘oncological’, ‘resisting'
	15	Mesothelin	‘mesothelin’, ‘mesothelioma’, ‘msln’, ‘pancreatic cancer’, ‘ovarian cancer’, ‘lung’, ‘maytansinoid’, ‘glycosylated’, ‘recombinant’, ‘over-express'
	18	TROP-2	‘trop2’, ‘glycoprotein’, ‘tacstd2’, ‘tumor targeting’, ‘TNBC’, ‘positive tumor’, ‘sacituzumab govitecan’, ‘bystander’, ‘vascular’, ‘permeability'
	21	FRα	‘folr1’, ‘folate receptor’, ‘folate’, ‘tumor growth’, ‘fra’, ‘glycosyl’, ‘igg1’, ‘ovarian cancer’, ‘chimeric antigen receptor’, ‘peritoneal cancer'
	24	AXL	‘axl’, ‘kinase inhibitor’, ‘angiogenesis’, ‘pbd’, ‘immune’, ‘mmae’, ‘mapk’, ‘melanoma’, ‘over-expression’, ‘gas6′
	28	CD73	‘cd73’, ‘adcc’, ‘immunoglobulin’, ‘extracellular domain’, ‘high-affinity’, ‘fc receptor’, ‘glycosylation site’, ‘engineered antibody’, ‘domain’, ‘over-expression'
	31	FAP	‘fap’, ‘fibroblast’, ‘stromal’, ‘vascular endothelial’, ‘endocytosis’, ‘polynucleotide’, ‘fusion’, ‘immunotoxin’, ‘analog’, ‘hinge region'
	35	PSMA	‘psma’, ‘prostate’, ‘prostate cancer’, ‘membrane antigen’, ‘prostate-specific’, ‘mipsagargin’, ‘internalization’, ‘single domain’, ‘radioligand’, ‘urea'
	36	TF	‘tissue factor’, ‘auristatin’, ‘tf’, ‘mmae’, ‘vedotin’, ‘fviia’, ‘cervical cancer’, ‘pancreatic adenocarcinoma’, ‘tisotumab’, ‘platinum-based'
Conjugation chemistry (15.4%)	0	Cysteine-based conjugation	‘cysteine’, ‘site-specific’, ‘engineered’, ‘bifunctional linker’, ‘carbonyl group’, ‘ror1’, ‘region’, ‘engineered cysteine’, ‘atom group’, ‘reacted'
	34	Carbonyl-amine coupling	‘carbonyl-nitrogen’, ‘amide’, ‘carbonyl group’, ‘nitrogen atom’, ‘carbamate’, ‘c-n', ‘n-terminal reaction’, ‘nucleophilic’, ‘atom group’, ‘terminus'
​	44	Covalent conjugation	‘covalent linker’, ‘covalent’, ‘radionuclide’, ‘bioconjugation’, ‘crosslinker’, ‘assembled’, ‘reactive’, ‘coupling chemistry’, ‘stability’, ‘maleimide'
	45	Transglutaminase-catalyzed conjugation	‘transglutaminase’, ‘sortase’, ‘enzymatic’, ‘microbial’, ‘mimetic’, ‘spacer’, ‘glutamine ', ‘glycine ', ‘peptide’, ‘acyltransferase'
Payload modality (15.1%)	6	Camptothecin-derived payloads	‘camptothecin’, ‘camptothecin derivative’, ‘payload’, ‘topo i', ‘reaction’, ‘exatecan’, ‘exatecan derivative’, ‘prodrug’, ‘analog’, ‘salt'
	7	Auristatin-based payloads	‘auristatin’, ‘auristatin analog’, ‘mmae’, ‘mmaf’, ‘monomethyl auristatin’, ‘dolastatin-10′, ‘tubulin inhibitor’, ‘cytotoxic’, ‘cd33’, ‘cd46′
	8	Peptide cytotoxins	‘cytotoxic’, ‘cyclic peptide’, ‘peptide’, ‘doxorubicin’, ‘amatoxin’, ‘a-amanitin’, ‘peptide receptor’, ‘tubulysin’, ‘protease-cleavable’, ‘polymerase'
	9	PBD payloads	‘benzodiazepine’, ‘pyrrolobenzodiazepine’, ‘pbd’, ‘pbd dimer’, ‘dimeric’, ‘crosslink’, ‘maleimide linker’, ‘warhead’, ‘glycol derivative’, ‘sulfur'
	19	Topo I inhibitor payloads	‘sn38’, ‘triple-negative breast’, ‘dxd’, ‘tnbc’, ‘parpi’, ‘microtubule inhibitor’, ‘metastatic’, ‘chemotherapy-resistant’, ‘non-small-cell lung’, ‘topo i'
	29	Maytansinoid payloads	‘maytansinoid’, ‘dm1’, ‘dm4’, ‘t-dm1’, ‘cytotoxic maytansinoid’, ‘disulfide linker’, ‘non-cleavable linker’, ‘microtubule inhibition’, ‘byproduct’, ‘coupled'
	32	Immunostimulatory payloads	‘immunogenicity’, ‘tlr7/8′, ‘cd73’, ‘b7h3’, ‘axl’, ‘gpc3’, ‘cldn18a2’, ‘p-cadherin’, ‘stimulation’, ‘activation'
	42	STING agonist payloads	‘sting agonist’, ‘agonistic’, ‘cgas’, ‘cdn’, ‘dinucleotide’, ‘albumin’, ‘single domain’, ‘immunoglobulin’, ‘cd22’, ‘fusion'
Biological mechanism (15.0%)	3	Immune checkpoint modulation	‘pd1’, ‘tigit’, ‘dll3’, ‘folr1’, ‘aptamer’, ‘scfv’, ‘anthracycline’, ‘immune-checkpoint’, ‘doxorubicin’, ‘melanoma'
	5	Interleukin immunocytokines	‘interleukin’, ‘il2’, ‘il2 receptor’, ‘il15’, ‘il10’, ‘cytokine’, ‘cd123’, ‘tnf-a', ‘fibronectin’, ‘tumor necrosis'
	12	Signaling pathway inhibition	‘agonist’, ‘kras’, ‘inhibitor’, ‘tyrosine-kinase inhibitor’, ‘agonistic’, ‘parp’, ‘signaling’, ‘modulator’, ‘selective’, ‘autoimmune'
	23	Anti-angiogenic signaling	‘vegf’, ‘angiogenic’, ‘angiogenesis’, ‘vegfr2’, ‘hyperproliferative’, ‘endothelial’, ‘agonistic’, ‘blood vessel’, ‘vascular’, ‘fibronectin'
	38	Interferon signaling modulation	‘interferon’, ‘interferon alpha’, ‘antagonist’, ‘neutralizing’, ‘ifnar’, ‘autoimmune’, ‘type-i', ‘transplant’, ‘integrin’, ‘immune-checkpoint'
	46	TME modulation	‘microenvironment’, ‘tumor microenvironment’, ‘immunecheckpoint inhibitor’, ‘vegf’, ‘pdl1’, ‘vascular endothelial’, ‘thioether’, ‘ctla-4′, ‘fibronectin’, ‘tnf'
Delivery system (4.3%)	10	Polymer-drug conjugates	‘polymeric’, ‘polymer-drug conjugate’, ‘ligand-drug conjugate’, ‘cleavable linker’, ‘polymer’, ‘isomer prodrug’, ‘enzyme-cleavable’, ‘heteroaryl group’, ‘gemcitabine’, ‘glycoprotein'
	16	Nanoparticle delivery	‘nanoparticle’, ‘liposome’, ‘nanobody’, ‘nanoparticle-drug conjugate’, ‘self-assembled’, ‘fab fragment’, ‘polysaccharide’, ‘lipid’, ‘multivalent’, ‘photosensitizer'
	26	Virus-like carriers	‘virus-like’, ‘viral’, ‘viral infection’, ‘phospholipid’, ‘liposome’, ‘peptide linker’, ‘virus’, ‘membrane’, ‘endosomal’, ‘fusion'
Therapeutic indication (4.2%)	20	HER2-positive breast cancer	‘her2-positive breast cancer’, ‘t-dm1’, ‘trastuzumab’, ‘pertuzumab’, ‘dm1’, ‘duocarmycin’, ‘taxane’, ‘anthracycline’, ‘metastatic’, ‘ihc'
	25	Multidrug resistance	‘multidrug’, ‘resistant’, ‘transporter’, ‘pd1/pdl1’, ‘pdl1/ctla4’, ‘c-met’, ‘overcome’, ‘traditional’, ‘tumor autoimmune’, ‘ph-responsive'
	27	Multiple myeloma	‘bcma’, ‘multiple myeloma’, ‘maturation’, ‘B cell’, ‘malignancy’, ‘B cell lymphoma’, ‘crosslinking’, ‘splicing’, ‘modulator’, ‘stabilized'
	33	Acute myeloid leukemia	acute myeloid-leukemia’, ‘cd33’, ‘myeloid leukemia antigen’, ‘aml’, ‘car’, ‘malignancy’, ‘transgenic’, ‘polynucleotide’, ‘hybridoma’, ‘inhibitor'
	40	B-cell lymphoma	‘cd19’, ‘B cell lymphoma’, ‘non-hodgkin lymphoma’, ‘B cell’, ‘malignancy’, ‘cd20’, ‘malignant’, ‘maytansinoid’, ‘lymphoid’, ‘hematologic'
Antibody architecture (3.0%)	14	Immune receptor engineering	‘chimeric-antigen-receptor’, ‘bispecific chimeric-antigen-receptor’, ‘bispecific antibody’, ‘cd40’, ‘activated’, ‘monoclonal antibody’, ‘immunoglobulin’, ‘cd4’, ‘sirp-a', ‘car'
	17	Antibody engineering	multispecific antibody’, ‘igg’, ‘bispecific antibody’, ‘fab’, ‘high-affinity’, ‘antigen specific’, ‘homogeneous’, ‘transglutaminase’, ‘n-terminus’, ‘c-terminus'
Linker chemistry (3.0%)	22	Dual-linker systems	‘bislinker’, ‘dual linker’, ‘click chemistry’, ‘disulfide linker’, ‘taxane’, ‘double linker’, ‘peptide linker’, ‘hydrazine’, ‘site-specific’, ‘alkylating'
	30	Hydrazone cleavable linkers	‘hydrazine’, ‘thiol’, ‘succinic’, ‘aldehyde’, ‘acid-sensitive’, ‘disubstituted’, ‘hydroxyl’, ‘homogeneous’, ‘hydrogen atom’, ‘acid'
	37	Self-immolative linkers	‘self-immolative linker’, ‘oxime’, ‘covalent linker’, ‘oxygen’, ‘hydrophobic’, ‘condensation’, ‘glucuronidase’, ‘trigger’, ‘side chain’, ‘nucleophilic'
	39	Thioether linkers	‘thioether’, ‘non-cleavable linker’, ‘smcc’, ‘thiol’, ‘mcc’, ‘n-maleimide’, ‘sulfur’, ‘tubulin inhibitor’, ‘substitution’, ‘cysteine'
Formulation and stability (1.1%)	41	ADC formulation optimization	‘trehalose’, ‘surfactant’, ‘sucrose’, ‘drug-to-antibody ratio’, ‘excipient’, ‘lyophilized’, ‘hydrophobic’, ‘auristatin’, ‘dxd’, ‘buffer'
	43	Surfactant-stabilized ADC formulations	‘surfactant’, ‘sugar excipient’, ‘sugar surfactant’, ‘freeze-drying’, ‘buffering’, ‘mannitol’, ‘scale-up’, ‘removal’, ‘polysorbate’, ‘stabilizer'

**FIGURE 5:**
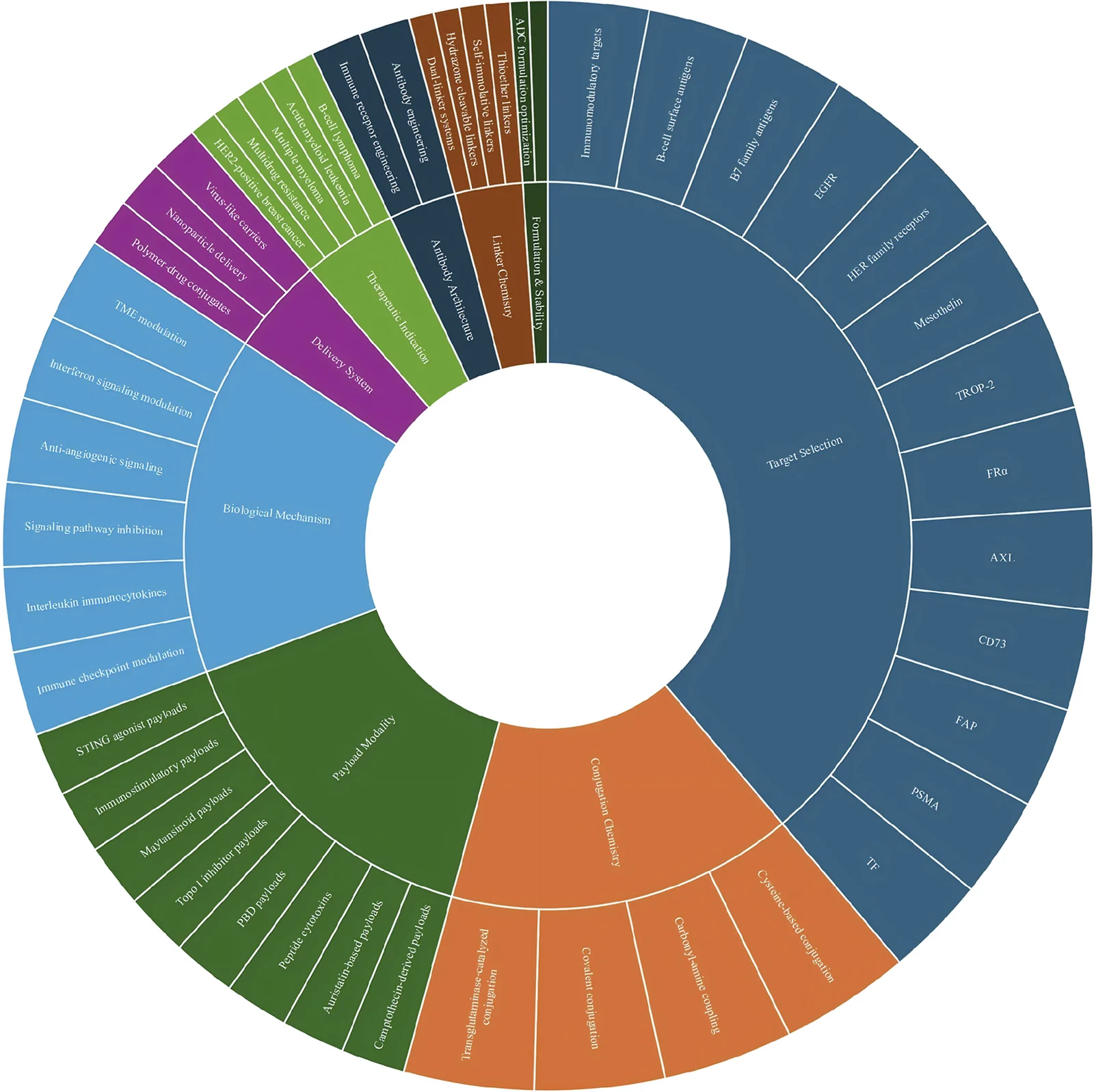
Hierarchical visualization of ADC technology topics. The inner ring represents the nine technological clusters, and the outer ring represents the 47 topics. Sector size is proportional to the number of patent families assigned to each topic. Different colors denote distinct technological clusters.

To complement the topic-level structure, Figure 6 summarizes the therapeutic targets, payloads, and linkers. Synonyms and abbreviations were consolidated into unified categories. Each patent family could contribute to multiple categories within the same panel, so the category proportions do not necessarily sum to 100%.

**FIGURE 6:**
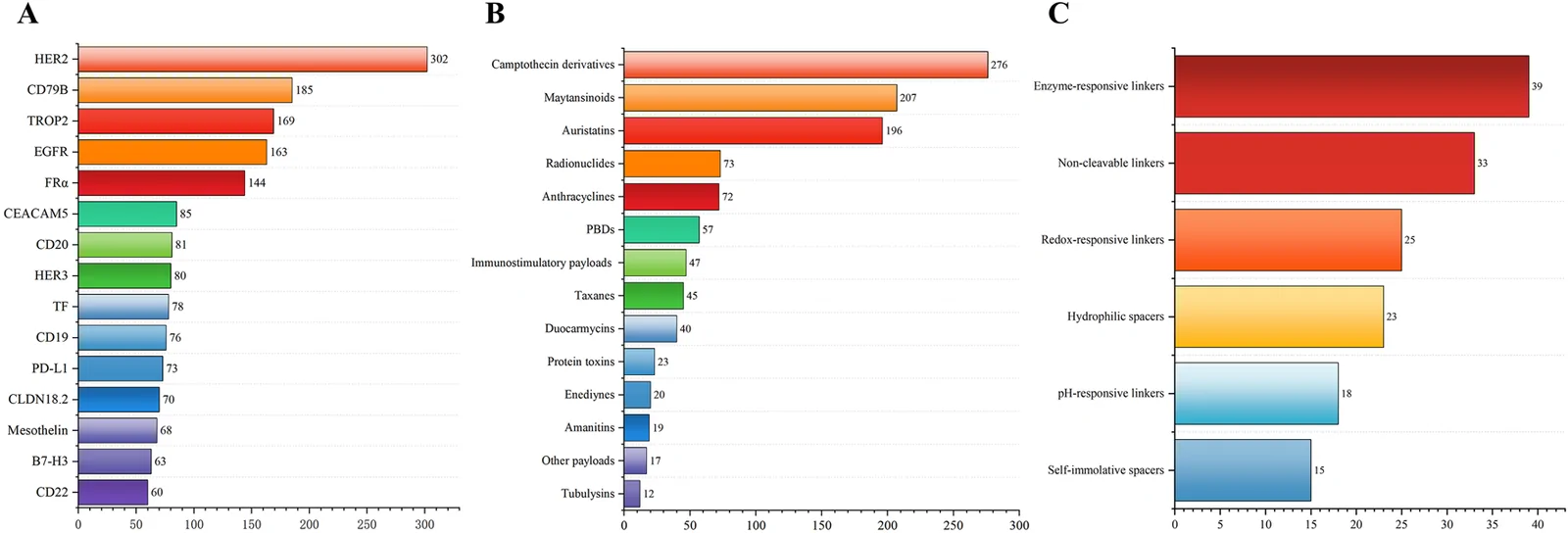
Distribution of ADC components. **(A)** targets; **(B)** payloads; and **(C)** linkers. Bar labels report the number and proportion of patent families in which each component was explicitly identified. Categories within each panel are non-exclusive.

#### 3.1.1 Biological module

##### 3.1.1.1 Target selection cluster

Target selection strongly affects therapeutic efficacy, toxicity window, and clinical tractability of ADCs (Beck et al., 2017). In the topic analysis, target-related topics constitute the largest cluster and centered on antigen druggability, expression patterns, and tissue distribution. Complementary with this topic-level structure, Figure 6A shows that HER2 was the most frequently represented therapeutic target (n = 302), followed by CD79b (n = 185), TROP-2 (n = 169), EGFR (n = 163), and FRα (n = 144). The leading targets include both hematologic antigens such as CD79b, and solid tumor antigens such as HER2, TROP-2, EGFR, and FRα, demonstrating the broad target diversity of the ADC patent landscape.

For hematologic malignancies, B-cell surface antigens (Topic 2) remain prominent because of lineage-specific expression. CD79b has been clinically validated by polatuzumab vedotin, which improved outcomes in relapsed/refractory diffuse large B-cell lymphoma (DLBCL) (Sehn et al., 2020). Some CD20-targeted ADCs have been constrained by insufficient internalization kinetics and lysosomal trafficking. Recent innovations has examined biparatopic designs and epitope selection strategies to improve endocytic uptake (Tarantelli and Bertoni, 2022; Dabkowska et al., 2024). Immune-related targets (Topic 1), represented by LAG-3, occupy a substantial proportion of this cluster, reflecting growing interest in immune regulation within the ADC target repertoire (Ackerman et al., 2021).

Solid tumor antigens account for a considerable share of topics, notably HER2 (Topic 13) and TROP-2 (Topic 18). The clinical success of HER2/DXd (T-DXd) (Modi et al., 2025) and TROP-2/SN-38 (SG) (Bardia et al., 2021) target-payload pairings, extended attention to low-to-intermediate expression, supported by high DAR and bystander active payloads (Ogitani et al., 2016). These findings have enriched attention to continuous antigen expression spectra in breast cancer, particularly the HER2 expression spectrum (Tarantino et al., 2022). EGFR (Topic 11) research mainly focuses on bispecific ADCs, including EGFR/HER3. Dual antigen binding may enhance tumor specificity and mitigate cutaneous toxicity caused by wild-type EGFR binding (Ma et al., 2024). Following the clinical validation of mirvetuximab soravtansine (MIRV), work on FRα (folate receptor; Topic 21) emphasized site-specific conjugation and DAR control to refine pharmacokinetics and minimize ocular toxicity (Moore et al., 2023). After the approval of tisotumab vedotin, work on TF (tissue factor; Topic 36) focused on coagulation-inert antibody deigns (Theunissen et al., 2018; De Bono et al., 2019) to decouple antitumor activity from Factor VIIa-mediated bleeding risk (Vergote et al., 2024).

The topic structure also highlights tumor microenvironment (TME)-associated targets. Targets such as B7 family (Topic 4), mesothelin (Topic 15), AXL (Topic 24), CD73 (Topic 28), FAP (fibroblast activation protein; Topic 31), and PSMA (prostate-specific membrane antigen; Topic 35) are increasingly distinguished from classical tumor cell markers. B7-H3 (CD276) has emerged as a pan-cancer target because it is overexpressed in malignant tissues and tumor vasculature (Seaman et al., 2017). Combination strategies with poly(ADP-ribose) polymerase (PARP) inhibitors are being explored to exploit DNA damage vulnerability (Cen et al., 2025). FAP-targeted strategies use FAP expression in cancer-associated fibroblast (CAFs)-rich stroma to deliver payloads into the stromal compartment, where bystander effect may affect neighboring tumor cells that are inaccessible to conventional agents (Desbois and Wang, 2021). PSMA-targeted ADCs and radioligands therapies have continued to progress in metastatic prostate cancer (Sartor et al., 2021). By targeting these TME-associated processes, ADCs may disrupt tumor-stromal crosstalk and increase intratumoral drug exposure in tumors less amenable to classical epithelial markers.

Overall, the Target Selection cluster shows an expanded ADC target space spanning hematologic lineage markers, pan-carcinoma antigens, and TME-associated targets.

##### 3.1.1.2 Biological mechanism cluster

The Biological Mechanism cluster complements target diversity by reorganizing around pathway intervention and immune regulation beyond binding affinity alone. It highlights the modulation of downstream signaling and the alteration of the TME by conjugates (Long et al., 2025). Immune checkpoint modulation (Topic 3) indicates that PD-1/PD-L1, DLL3, CTLA-4, and related molecules are positioned within coordinated inhibitory circuits. Patent documents in this area frequently pair immune activating payloads with checkpoint targeting antibodies (Ackerman et al., 2021). Such designs can relieve inhibitory signaling and deliver innate immune stimulation to myeloid cells, which may introduce immune activity into immunologically cold tumors (Fong et al., 2025). Interleukin (IL) immunocytokines (Topic 5) fuse IL-2 or IL-15 with tumor targeting antibodies, concentrating cytokine activity at tumor site and supporting local expansion of effector and memory lymphocytes. This design may reduce systemic toxicity observed with high-dose cytokine therapy (Agliardi et al., 2025). Topics related to interferon signaling modulation (Topic 38) and TME modulation (Topic 46) further apply this logic by using conjugates to influence innate immune tone, stromal activation, and vascular permeability within the tumor bed (Kundu et al., 2024).

Beyond immune modulation, this cluster shows the integration of conjugate strategies with classical oncogenic signaling axes, where antibodies contribute target recognition and pathway intervention. Signaling pathway (Topic 12) and anti-angiogenic signaling (Topic 23) exemplify this pattern, in which conjugate designs are combined with pathway inhibition, including KRAS/RTK or PARP. Systemic dosing of these agents is often limited by off-target toxicity (Casi and Neri, 2015). In VEGF/VEGFR-targeted therapies, novel conjugates use tumor vasculature as a docking site for payload delivery to endothelial cells (Wang D. et al., 2022). This approach can block tumor blood supply and induce direct vascular injury, intensifying metabolic stress in the tumor core (Gerber et al., 2009).

The Biological Mechanism cluster suggests that ADC design increasingly combines payload delivery with immune and signaling control. This approach may address tumor heterogeneity and drug resistance through immune activation and pathway blockade, connecting Target Selection with Therapeutic Indication.

##### 3.1.1.3 Antibody architecture cluster

Although the Antibody Architecture cluster accounts for a relatively small share of patent families, the expansion of targets and mechanisms still relies on molecular redesign of antibody structures. This cluster focuses on full-length immunoglobulins and connects targets recognition with mechanism modulation by altering antibodies and receptors (Riccardi et al., 2023). Antibody engineering (Topic 17) centers on reconfiguring conventional IgG scaffolds into bispecific and multispecific formats to enhance target selectivity and internalization. A critical innovation in this domain is the development of biparatopic antibodies that recognize two non-overlapping epitopes on a single antigen, such as HER2 or MET. These constructs promote receptor clustering and crosslinking on the tumor cell surface, drive rapid internalization and lysosomal trafficking, and improve the druggability of targets with poor internalization efficiency (Li J. Y. et al., 2016). When combined with controlled conjugation strategies, these designs can enhance structural homogeneity and DAR control while optimizing antigen engagement and internalization under heterogeneous target expression. Emerging bispecific ADCs exemplify this trend by improving tumor specificity, internalization, and payload delivery through dual antigen recognition within a defined linker-payload system (Gu et al., 2024a).

Immune receptor engineering (Topic 14) applies antibody-derived modular design principles to engineered receptor systems. This topic reflects the transfer of antibody engineering principles to receptor-based therapeutic systems, particularly chimeric antigen receptor (CAR) platforms. These constructs can amplify or redirect immune effector activity by combining modular binding domains, such as single-chain variable fragments (scFvs) or antigen-binding fragments (Fabs), with flexible linkers and immune activating or checkpoint-modulating units in the TME. These systems may enhance antigen-dependent activation, phagocytosis, or costimulation in engineered T cells or myeloid cells (Liu Y.e. et al., 2025a).

The Antibody Architecture cluster provides a structural basis for expanded target and mechanism spaces. Through antibody and receptor format redesign, these topics demonstrate an evolution toward multi-input recognition systems that better match heterogeneous tumor biology.

##### 3.1.1.4 Therapeutic Indication cluster

The Therapeutic Indication cluster delineates ADC technologies around clinically important disease settings. HER2-positive breast cancer (Topic 20) is anchored in HER2, which also occupies a core position in the Target Selection cluster. Patents under this topic typically combine antibodies targeting HER2 with maytansinoid or Topo I inhibitor payloads and optimized linker chemistry to address heterogeneous HER2 expression, including HER2-low disease. The remaining indication topics primarily focus on hematologic malignancies. Multiple myeloma (Topic 27) and B-cell lymphoma (Topic 40) indicate close associations between lineage-restricted antigens, such as B-cell maturation antigen (BCMA), CD19, and CD20, and payload-linker combinations optimized for antigen density and internalization. In BCMA-directed ADCs for multiple myeloma, efficacy must be balanced against ocular toxicity, particularly keratopathy, as represented by mafodotin-containing conjugates (Lonial et al., 2020). Acute myeloid leukemia (AML; Topic 33) is organized around CD33 and other AML antigens, with indication pattern shaped by myeloid lineage biology, hematopoietic toxicity, and dose-response balance (Walter et al., 2012). Unlike these disease defined topics, multidrug resistance (Topic 25) captures a cross disease resistance phenotype across tumor types. Here, ADCs are positioned as tools to address transporter mediated resistance, such as P-glycoprotein efflux, and microenvironment mediated tolerance (Št’astný et al., 1999). By delivering cytotoxic payloads intracellularly via receptor-mediated endocytosis, these conjugates may reduce reliance on uptake pathways affected by membrane efflux pumps.

Consistent with the Biological Mechanism cluster, this pattern indicates that the Therapeutic Indication cluster increasingly captures composite clinical features, including expression heterogeneity, resistance phenotypes, lineage biology, and toxity constraints, in addition to target disease labels.

#### 3.1.2 Chemical module

##### 3.1.2.1 Conjugation chemistry cluster

At the chemical implementation level, the Conjugation Chemistry cluster defines payload attachment to antibodies, influencing DAR distribution, conjugate homogeneity, and payload retention in circulation. Cysteine-based conjugation (Topic 0) covers both interchain disulfide and engineered cysteine strategies. Interchain conjugation proceeds through partial reduction of endogenous disulfide bonds, followed by maleimide–thiol coupling. Compared with early lysine-based random conjugation, this approach operates under milder conditions and yields narrower DAR distributions, although retro-Michael deconjugation and premature payload loss *in vivo* remain concerns (Ponte et al., 2016). Engineered cysteine approaches, represented by THIOMAB technology, introduce cysteine residues at predefined antibody positions and site-specific conjugation with improved homogeneity and *in vivo* stability when the engineered sites preserve antigen binding and Fc-mediated functions (Junutula et al., 2008). These approaches demonstrate that precise control over conjugation sites can separate drug loading from random surface reactivity, supporting a wider therapeutic window.

Transglutaminase-catalyzed conjugation (Topic 45) provides an enzymatic route to site-specific attachment. In this approach, glutamine or lysine motifs recognized by microbial transglutaminase are engineered into the antibody backbone, and payloads are coupled to these sites through enzymatic catalysis. The resulting ADCs are highly homogeneous and have well-defined drug loading. This precision, however, may introduce process complexity and potential immunogenicity risks associated with non-native peptide sequences or residual bacterial enzymes (Dennler et al., 2014). Carbonyl-amine coupling (Topic 34), including amide and carbamate architectures, is valued for plasma hydrolytic stability and more predictable degradation under lysosomal conditions. These linkage are often contrasted with ester or carbonate linkers, which can show faster cleavage and less controlled release (Alas et al., 2020). Covalent conjugation (Topic 44) covers general bioconjugation chemistries, such as maleimides and bifunctional crosslinkers. Patents in this topic balance coupling efficiency with *in vivo* robustness, for example, by stabilizing thiosuccinimide intermediates or promoting maleimide hydrolysis after conjugation to suppress payload transfer and improve conjugate stability (Wang Y. et al., 2022).

##### 3.1.2.2 Linker chemistry cluster

The Linker Chemistry cluster refines the bond types and trigger units that govern payload retention and release after conjugation. This cluter is organized around environment-responsive and enzyme triggered release strategies.

The linker chemical cluster encompasses the release mechanism and spacer base characteristics, which regulate the retention, solubility, and activation of the payload after conjugation. Figure 6C shows that enzyme-responsive linkers are the most commonly selected (n = 39), followed by non-cleavable linkers (n = 33), redox-responsive linkers (n = 25), hydrophilic spacer (n = 23), pH-responsive linkers (n = 18), and self-cleavable spacer (n = 15).

Hydrazone cleavable linkers (Topic 30) represent an early generation strategy that exploits the pH difference between physiological conditions (pH 7.4) and acidic endosomal or lysosomal compartments (pH 4.5–6.0). These acid-labile hydrazone or acetal bonds promote hydrolysis in acidic intracellular compartments, while insufficient systemic stability can lead to nonspecific drug release in circulation (Tsuchikama and An, 2018). To address systemic stability, thioether linkers (Topic 39) represent non-cleavable designs, exemplified by succinimidyl SMCC-based structures. In these designs, the linker remains intact, and active catabolites are generated after lysosomal antibody degradation. The resulting payload-linker-amino acid catabolites exhibit high plasma stability and are typically charged, with limited membrane permeability. This profile weakens bystander effect and may limit efficacy in tumors with heterogeneous antigen expression (Fu et al., 2022). Self-immolative linkers (Topic 37) combine systemic stability with the release of unmodified, membrane-permeable payloads. These designs incorporate a cleavable trigger, such as a protease-sensitive peptide (Val-Cit) or β-glucuronidase-sensitive unit, coupled to a self-immolative spacer such as p-aminobenzyl carbamate (PABC). Upon enzymatic activation in lysosomes, the spacer undergoes a self-immolative 1,6-elimination cascade, releasing the payload in its active form while limiting premature payload exposure during circulation (Edupuganti et al., 2021). Dual-linker systems (Topic 22) explore bis-linker formats that integrate cleavable and non-cleavable units or distinct cleavable triggers. Such structures tune DAR, payload stoichiometry, and release kinetics, and may deliver two payloads with complementary mechanisms within the same conjugate (Khosravifarsani et al., 2025).

The Conjugation Chemistry and Linker Chemistry clusters indicate more controlled design of payload attachment, bond types, and activation triggers. These clusters support modular, environment responsive linker structures aligned with specific targets, mechanisms, and indications.

##### 3.1.2.3 Payload modality cluster

The Payload Modality cluster is characterized by the coexistence of established cytotoxic payloads and newer immune active modalities. Figure 6B shows that camptothecin derivatives were the most frequently payload category (n = 276), followed by maytansinoids (n = 207) and auristatins (n = 196) Their simultaneous prominence indicates that topoisomerase I inhibitor platform coexists with the established microtubule-targeting maytansinoid and auristatin technologies in the current ADC patent landscape.A stable group centers on classical microtubule inhibitors, specifically maytansinoid payloads (Topic 29) and auristatin-based payloads (Topic 7). Based on decades of clinical experience with agents such as trastuzumab emtansine (T-DM1) and polatuzumab vedotin, innovation in this domain has focused on linker-payload interface optimization. Current patenting activity emphasizes hydrophobicity and DAR adjustment to balance bystander activity, intracellular retention, and systemic tolerability, with the aim of reducing peripheral neuropathy and ocular toxicity associated with MMAE and DM4 (Li F. et al., 2016b).

To address heterogeneous solid tumors and multidrug resistance, DNA-targeting payloads have gained prominence. This category includes pyrrolobenzodiazepine (PBD) payloads (Topic 9) and camptothecin-derived payloads (Topic 6), and broader Topo I inhibitor payloads (Topic 19). PBD dimers exhibit picomolar potency suitable for low-expression targets, however, their narrow therapeutic window limits broad application (Hartley, 2021). Topo I inhibitors, including SN-38 and DXd, provide an alternative with improved tolerability. These designs employ moderately potent payloads, allowing for high DAR of approximately 8 and strong membrane permeability, generating bystander cytotoxicity and potentially eliminating antigen-low or antigen-negative tumor cells in heterogeneous tumors (Pommier and Thomas, 2023). Peptide cytotoxins (Topic 8) include new mechanisms such as macrocyclic peptides (e.g., amatoxins), which inhibit RNA polymerase II and may retain activity against tumors resistant to microtubule inhibitors and DNA-damaging agents (Orlik et al., 2022).

Consistent with the Target Selection cluster, ADC innovation increasingly addresses solid tumors in addition to hematologic malignancies. Payload preferences favor DNA- and RNA-directed cytotoxic agents, together with payloads functions beyond direct cell killing. Immunostimulatory payloads (Topic 32) and stimulator of interferon genes (STING) agonist payloads (Topic 42) incorporate Toll-like receptor (TLR) agonists or STING pathway activators into ADCs. These immune-stimulating antibody conjugates (ISACs) can kill target cells, promote tumor antigen release, induce proinflammatory signaling, which may convert cold tumors into hot tumors and connect targeted chemotherapy with immunotherapy (Ackerman et al., 2021). In summary, payload modalities transform target selection into specific functional outputs. They have evolved ADC platform development capable of modulating the TME (Fang et al., 2025), in alignment with the immune pathways highlighted in the Biological Mechanism cluster.

The Payload Modality cluster indicates that payload design systematically optimizes microtubule- or DNA-targeted payloads through linker design and DAR engineering. Additionally, by incorporating RNA targeting and immunostimulatory payloads, ADC function is extended to immune and microenvironmental modulation. Within the overall topic framework, this cluster translates upstream decisions regarding targets, mechanisms, antibody formats, and conjugation and linker chemistry into functional outputs (Conilh et al., 2023).

#### 3.1.3 System module

In addition to biological and chemical modules, system-related clusters outline the technological requirements for converting ADC molecular designs from into controllable delivery systems and manufacturable drug products.

##### 3.1.3.1 Delivery system cluster

The Delivery System cluster addresses the pharmacokinetic and tissue distribution limitations of conventional IgG carriers. It covers polymer-drug conjugates (Topic 10), nanoparticle delivery (Topic 16), and virus-like carriers (Topic 26). Patents in these topics introduce polymeric micelles, liposomes, and inorganic nanoparticles as delivery carriers to prolong circulation half-life and improve local payload release (Ekladious et al., 2019). A key innovation is antibody-conjugated polymeric micelles that concentrate hydrophobic payloads within a nanoscale core and present antibodies on the corona (Junyaprasert and Thummarati, 2023). This architecture limits premature payload degradation and uses the enhanced permeability and retention (EPR) effect to support tumor accumulation (Abdelhamid et al., 2025). Virus-like particles or virus-inspired assemblies package active compounds within organized protein shells and may enhance cellular trafficking and tissue penetration (Ikwuagwu and Tullman-Ercek, 2022).

##### 3.1.3.2 Formulation and stability cluster

The Formulation and Stability cluster, despite representing the smallest share of documents, addresses critical challenges in industrial manufacturing and clinical use. It involves lyophilization, selection of sugars and surfactants, buffer systems, and pH control (Frka-Petesic et al., 2016). ADCs are prone to aggregation because of hydrophobic payload attachment. Therefore, formulation optimization (Topic 41) focuses on maintaining DAR distribution, colloidal stability, and chemical integrity (Wakankar et al., 2011). Payloads attachment can reduce antibody solubility, create hydrophobic regions that promote aggregation, increase free drug levels through deconjugation, and destabilize critical quality attributes under thermal, mechanical, or interfacial stress (Strop et al., 2013). These studies show that conjugation can exacerbate aggregation, fragmentation, and payload loss compared with unconjugated antibodies (Beckley et al., 2013).

Research on surfactant-stabilized formulations (Topic 43) highlights the role of surfactants such as polysorbates. Polysorbates can protect against interfacial stress, while their oxidative and hydrolytic degradation may generate peroxides and reactive aldehydes that compromise payload integrity, antibody structure, and long-term stability (Kishore et al., 2011). The cluster defines ADCs as structurally complex biologic products and positions dosage form engineering as part of ADC technology by connecting molecular structure, delivery behavior, and manufacturability.

### 3.2 Patent citation landscape

#### 3.2.1 Patent citation network structure

The extracted giant component of the citation network reveals an active knowledge exchange structure with 901 patent family nodes and 847 directed edges (Figure 7) (Newman, 2003). Among these nodes, 628 families had nonzero out-degree, indicating reliance on prior patent knowledge, and 373 had nonzero in-degree, reflecting recognition by subsequent inventions. This numerical disparity suggests that most patent families draw on existing technologies, whereas only a restricted subset becomes recurrent reference points for downstream R&D. This pattern characterizes the recombinant nature of technological evolution, in which knowledge integration coexists with knowledge diffusion (Jaffe and de Rassenfosse, 2017).

**FIGURE 7:**
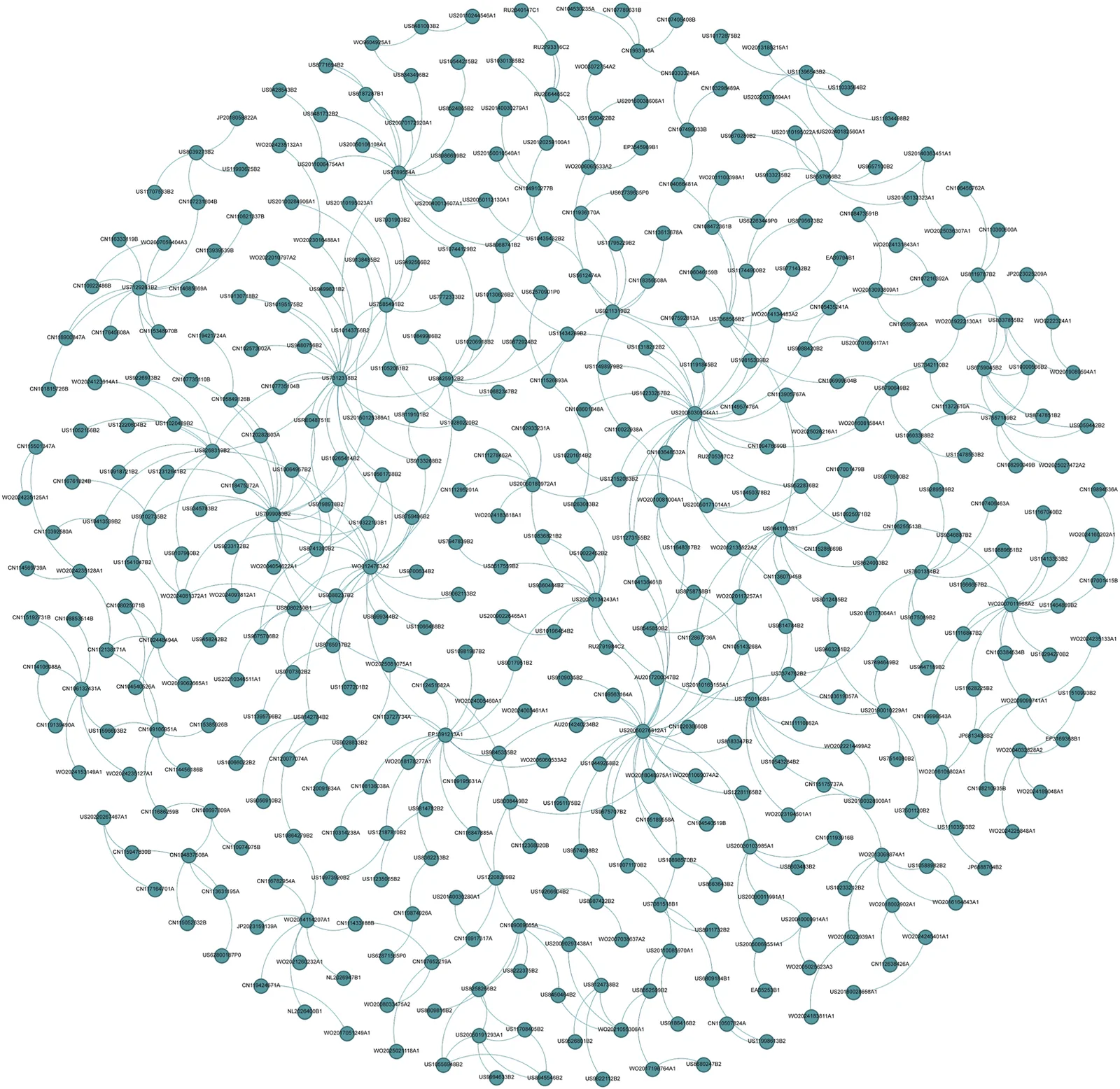
Patent citation network.

The cross-degree metric was applied with λ=0.5 . Patent families with high cross-degree occupy central structural positions in the citation network and connect multiple technological streams. These high-impact patents concentrate citation flow and shape dominant technological trajectories in ADC development (Newman, 2018). The metric distributions demonstrates that in-degree was more skewed than out-degree, with maximum values of 24 and 8, respectively. Consequently, the cross-degree ranking more closely follows in-degree, meaning that highly cited families were more likely to occupy top positions. Nodes characterized mainly by high out-degree rarely reached leading cross-degree positions, highlighting that downstream citation recognition carried disproportionate weight. This skewness reflects the hierarchical structure of the patent knowledge network.

To further distinguish modes of knowledge flow, structurally significant patents were separated into high-impact patents and influential patents based on topological features. High-impact patents were defined by high cross-degree, indicating strong capacity for both knowledge absorption and dissemination. Influential patents refer to patents with outstanding performance along a single dimension, either in-degree or out-degree. Within this classification, high in-degree patents represent concentrated knowledge disseminators that establish foundational platforms. High out-degree patents represent intensive knowledge absorbers that aggregate prior art to optimize specific technical domains.

#### 3.2.2 High-impact patents

As listed in Table 2, 15 high-impact patents were identified. The patent with the highest cross-degree was US7999083B2 (Govindan et al., 2009), which describes a hydrolyzable CL2A-SN-38 linker-payload system. In this disclosure, SN-38 is functionalized at the 20-hydroxyl position via a carbonate bond that preserves the active lactone ring and is coupled to antibodies through a pH-sensitive CL2A linker containing L-amino acids. This design generates SN-38 immunoconjugates, such as Mab-CL2A-SN-38, that can be paired with antibodies targeting multiple tumor-associated antigens (TAAs). Four additional high-impact patents also concentrate on the CL2A-SN-38 platform. US7585491B2 (Govindan, 2009a) details a modular linker that combines amino acid residues with thiol-reactive groups and connects ester bond cleavage with intracellular release after antibody internalization. It also discloses a conjugation strategy that reduces interchain disulfides to generate an average of eight free thiols, facilitating reproducible multisite conjugation. US8425912B2 (Govindan, 2013) and US8268319B2 (Govindan, 2009b) extend intracellular cleavable linker strategies to autoimmune diseases and malignant tumors, respectively, and specifically mention camptothecins like CPT-11 (irinotecan) and SN-38. These patents select target repertoires, including B-cell lineage antigens, myeloid antigens, EpCAM, and HER-2/neu, for multivalent and multispecific antibody formats. The linker is positioned as reusable delivery chemistry across diverse indications and antigens.

**TABLE 2:** High-impact patents of ADC.

Rank	Cross-degree	In-degree	Out-degree	Patent no.	Assignee	Representative technologies	Tech focus (topic/Cluster)
1	6.55	18	2	US7999083B2	Gilead Sciences	Hydrolyzable SN-38 antibody conjugation platform	Topic 6/Payload modality
2	6.07	24	1	US20050276812A1	Genentech/Roche	HER2-targeted maytansinoid conjugates based on trastuzumab-derived antibodies	Topic 29/Payload modality
3	5	8	3	US8425912B2	Gilead Sciences	Intracellularly cleavable camptothecin immunoconjugate platform	Topic 6/Payload modality
4	4.66	15	1	EP1391213A1	Boehringer Ingelheim	Combination therapy of CD44v6-targeted maytansinoid conjugates for solid tumors	Topic 29/Payload modality
5	4.20	8	2	US8268319B2	Gilead Sciences	Camptothecin immunoconjugate platform with intracellularly cleavable linkers	Topic 6/Payload modality
6	4	24	0	US20080305044A1	Seagen	Site-specific cysteine conjugation for auristatin ADCs with controlled DAR	Topic 7/Payload modality
7	3.58	6	2	US7368565B2	Immuno-Gen	One-step preparation of maytansinoid cytotoxic conjugates via disulfide linkers	Topic 29/Payload modality
8	3.58	6	2	US9211319B2	Seagen	Weekly BV dosing regimen of anti-CD30 VC-PAB-MMAE	Topic 7/Payload modality
9	3.47	19	0	WO0124763A2	Immuno-Gen	Combination therapy of immunoconjugates and chemotherapeutics for cancers	Topic 17/Antibody architecture
10	3.47	19	0	US7312318B2	Immuno-medics	CD74-targeted antibodies and immunoconjugates for tumors and autoimmune diseases	Topic 2/Target selection
11	3.47	9	1	US20070134243A1	Genentech/Roche	Val-Cit-PAB linker platform for intracellular release of DNA-binding payloads	Topic 37/Linker chemistry
12	3.24	8	1	US7585491B2	Gilead Sciences	Thiol-based multisite antibody conjugation via interchain disulfide reduction	Topic 0/Conjugation chemistry
13	3.24	8	1	US7750116B1	Seagen	VC-MMAE ADCs as metabolically activated auristatin prodrugs	Topic 7/Payload modality
14	3.24	8	1	US8080250B1	Gilead Sciences	Amino acid ester-based SN-38 scaffold with solubility, stability, and release	Topic 6/Payload modality
15	3.24	8	1	US8557966B2	Immuno-Gen	FRα-targeted DM4 antibody conjugates with controlled DAR and stable linker design	Topic 21/Target selection

US8080250B1 (Govindan et al., 2011) discloses synthetic routes for multiple linker variants CL2A, CL6, CL7, CLX, and CLY, by modifying hydrophilic COR substituents. These variants adjust the solubility, plasma stability, and intracellular release of SN-38 conjugates. This series of high-impact SN-38/CL2A patents establishes an amino acid ester-based linker-payload scaffold and optimization strategies for Topo I inhibitor ADCs. SG covalently couples SN-38 to a humanized anti-TROP-2 IgG1 antibody through the hydrolyzable CL2A linker, with an average DAR of approximately 7–8 (Tolaney et al., 2025), exemplifies the clinical relevance of this platform. Clinical evidence indicates that SG enhances progression-free survival and overall survival in mTNBC compared with conventional monochemotherapy (Bardia et al., 2021).

High-impact patents related to targets define biological and antibody engineering foundations for conjugate development. US7312318B2 (Hansen et al., 2007), assigned to Immunomedics Inc., focuses on CD74 antibodies and their derivatives, including humanized, chimeric, and fully human monoclonal antibodies. It also covers antibody fusion proteins, immunoconjugates, vaccines, and bispecific molecules for treating and diagnosing CD74-positive tumors and autoimmune diseases. US8557966B2 (Ab et al., 2013) claims antibodies that specifically bind FRα and are covalently coupled to potent payloads, including DM4, via cleavable thiol linkers such as sulfo-SPDB or SMCC. This molecular design achieved an average DAR of 3–4 while maintaining plasma stability and supporting the clinical translation of MIRV, an approved FRα-targeted ADC.

A group of high-impact patents focuses on maytansinoid payload technologies. US7368565B2 (Chari and Widdison, 2008), assigned to Immunomedics Inc., discloses a simplified preparation process in which maytansinoids bearing N-methylalanine or N-methylcysteine moieties and disulfide-reactive ester groups are coincubated with antibodies under mild conditions. This process supports efficient production of maytansinoid ADCs while preserving intracellularly cleavable disulfide bonds. US20050276812A1 (Ebens et al., 2005), assigned to Roche/Genentech, details HER2-targeted maytansinoid conjugates in which humanized 4D5 variants and related ErbB-recognizing antibodies are conjugated to DM1 and other maytansinoid cytotoxins through cysteine residues and non-disulfide linkers. The disclosure provides monotherapy and combination regimens for HER2-overexpressing breast cancer and other solid tumors. Genentech, a Roche subsidiary, obtained an exclusive license from ImmunoGen for DM1 in HER2-positive tumors, supporting the subsequent approval of T-DM1. T-DM1 was the first ADC approved worldwide for treating solid tumors (Sato et al., 2024). EP1391213A1 (Adolf et al., 2004) extends maytansinoid applications to CD44v6-high solid tumors, with approximately 3–4 maytansinoid residues per antibody. It also describes conjugation with microtubule-binding chemotherapy regimens based on taxanes, epothilones, and vinca alkaloids.

At the payload-linker level, US20070134243A1 (Gazzard et al., 2005) discloses 1,8-bis-naphthalimide derivatives as DNA-interacting cytotoxic motifs. These derivatives modulate physicochemical properties relevant to intracellular delivery, including molecular hydrophilicity, basicity, and DNA-binding affinity. Protease-cleavable peptide linkers assemble extension units, amino acid fragments, and spacer arms to ensure antigen-mediated endocytosis and intracellular release.

In addition, three high-impact patents assigned to Seagen Inc, concern auristatin ADC technologies. US20080305044A1 (McDonagh and Carter, 2008) introduces site-specific cysteine substitutions near interchain disulfide bonds in IgG1/IgG4 antibodies to predefine small-molecule conjugation sites. This approach yields ADCs with controlled DAR and conjugation configurations while preserving antigen-binding activity. The patent uses MMAE and MMAF as representative auristatin payloads and applies them to multiple TAAs, including CD20, CD30, CD33, CD40, CD70, and Lewis Y. Seagen developed brentuximab vedotin (BV), the first CD30-directed ADC, which exemplifies this cysteine conjugation strategy. It couples a chimeric anti-CD30 IgG1 antibody to the microtubule inhibitor MMAE through an MC-Val-Cit-PABC linker. US7750116B1 (Doronina and Alley, 2010) characterizes VC-MMAE ADCs as metabolically activated prodrugs. For auristatin ADCs with Val-Cit linkers and cysteine conjugation, the patent isolates and characterizes lysosomal metabolites dominated by cysteine-linker-drug conjugates.These metabolites retain substantial cytotoxicity in refractory or drug-resistant tumor models. US9211319B2 (Sievers et al., 2015) defines clinical dosing regimens for BV. It reports that weekly monotherapy at 0.8–1.2 mg/kg achieved a favorable balance between efficacy and tolerability in Hodgkin lymphoma and anaplastic large cell lymphoma.

Among high-impact patents related to therapeutic strategy, WO0124763A2 (Chari, 2001) proposes combined regimens of immunoconjugate and chemotherapeutic agents from a dosing perspective. The treatment uses humanized monoclonal antibodies or fragments targeting TAAs, including CD56-targeted humanized N901 and C242. It combines immunoconjugates carrying antimitotic agents such as DM1, maytansinoid analogues, vinca alkaloids, camptothecin, and Topo I inhibitors, with chemotherapeutic drugs for treating solid tumors and certain hematologic malignancies. The combination strategy was also extended to autoimmune diseases, transplant rejection, and viral or parasitic infections.

#### 3.2.3 Influential patents

As shown in Table 3, influential patents were identified through high in-degree or high out-degree in the patent citation network. The high in-degree patents listed in Table 3A provide crucial references for subsequent inventions. Among the ten influential patents with high in-degree, four influential patents were remained after excluding entries overlapping with high-impact patents. US6441163B1 (Chari and Widdison, 2002) discloses a one-step process for preparing maytansinoid conjugates with cel binding agents. The maytansinoid derivatives contain disulfide bonds and reactive N-sulfosuccinimidyl ester, which can directly couple with antibodies under mild conditions without prior protein modification. The disulfide linkage preserves intracellular cleavage required for cytotoxicity. In antibody engineering, US5789554A (Leung and Hansen, 1998) details chimeric and humanized LL2 antibody variants that retain the binding specificity and internalization efficiency of the parental murine anti-B-cell lymphoma antibody while reducing human anti-mouse antibody responses. The disclosure further describes LL2 conjugation with cytotoxic agents or markers for treating and diagnosing B-cell lymphoma and leukemia.

**TABLE 3:** Influential patents of ADC A. Influential patents with high in-degree (knowledge contributors).

Rank	Also in high-impact	In-degree	Out-degree	Patent no.	Assignee	Representative technologies	Tech focus (topic/Cluster)
1	Yes	24	1	US20050276812A1	Genentech/Roche	HER2-targeted maytansinoid conjugates based on trastuzumab-derived antibodies	Topic 29/Payload modality
2	Yes	24	0	US20080305044A1	Seagen	Site-specific cysteine conjugation for auristatin ADCs with controlled DAR	Topic 7/Payload modality
3	Yes	19	0	WO0124763A2	ImmunoGen	Combination therapy of immunoconjugates and chemotherapeutics for cancers	Topic 17/Antibody architecture
4	Yes	19	0	US7312318B2	Immuno-medics	CD74-targeted antibodies and immunoconjugates for tumors and autoimmune diseases	Topic 2/Target selection
5	Yes	18	2	US7999083B2	Gilead Sciences	Hydrolyzable SN-38 antibody conjugation platform	Topic 6/Payload modality
6	Yes	15	1	EP1391213A1	Boehringer Ingelheim	Combination therapy of CD44v6-targeted maytansinoid conjugates for solid tumors	Topic 29/Payload modality
7	No	14	0	US5789554A	Immuno-medics	Humanized LL2 monoclonal antibodieswith retained internalization and reduced immunogenicity	Topic 17/Antibody architecture
8	No	13	0	WO2007011968A2	Seagen	β-glucuronide self-immolative linker for selective intracellular payload release	Topic 37/Linker chemistry
9	No	11	0	US7129261B2	E. R. Squibb and Sons	Cleavable linkers for stabilized duocarmycin prodrug delivery intracellular release	Topic 37/Linker chemistry
10	No	10	0	US6441163B1	ImmunoGen	One-step lysine conjugation of disulfide-cleavable maytansinoid payloads	Topic 44/Conjugation chemistry

As linker technologies became more complex, US7129261B2 (Ng et al., 2006) introduced a prodrug platform for the potent DNA alkylating agent dacarbazine. This invention provides a general design for systemic delivery of masked payloads that are activated within target cells through peptide- or disulfide-based cleavable linkers. WO2007011968A2 (Jeffrey, 2007) incorporates a β-glucuronidase responsive unit into the linker-drug structure. Enzymatic cleavage supports selective release through lysosomal glycosidase activity after internalization and reduces aggregation associated with hydrophobic payloads, providing a reference for enzyme-sensitive ADCs.

High out-degree patents indicate intensive knowledge absorption and often represent later-stage optimization of existing platforms or adaption to industrial-scale production. The high out-degree influential patents identified listed in Table 3B showed no overlap with high-impact patents, yet several continued to track CL2A and camptothecin. US9198978B2 (Govindan et al., 2015) extends camptothecin conjugation chemistry through a modular two-step coupling strategy. This approach pre-synthesizes drug-linker intermediates containing short PEG spacer units and cleavable carbonate esters before Cu(I)-catalyzed azide-alkyne click coupling. This strategy resolves solubility and lactone instability issues in CPT-11/SN-38 derivatives and supports stable conjugates with high DAR (7–12). WO2024081372A1 (Calimsiz et al., 2024) focuses on process optimization for SG and labetuzumab govitecan. Refinement of intermediate purification and antibody reduction conditions improves homogeneity, with DAR maintained at 7–8 during scale-up production. Complementing process improvements, WO2021055306A1 (Kotapati et al., 2021) establishes a dual-capture assay for monitoring chemical transformations of ADC partner molecules while they remain attached to antibody fragments.

**TABLE 3B:** . Influential patents with high out-degree (knowledge absorbers).

Rank	Also in high-impact	In-degree	Out-degree	Patent no.	Assignee	Representative technologies	Tech focus (topic/Cluster)
1	No	0	8	US8034905B2	OncXerna therapeutics	Anti-VEGF antibodies selectively block VEGFR2 for treating angiogenic diseases	Topic 6/Biological mechanism
2	No	0	7	CN109069665A	Bristol-myers Squibb	Stabilized maleimide thioether linker for tubulysin ADCs with preserved lysosomal cleavage	Topic 39/Linker chemistry
3	No	0	7	US20230183326A1	Asana biosciences	Polymer scaffolds for decoupling payload capacity from antibody hydrophobicity	Topic 10/Delivery system
4	No	0	6	US7153932B2	Research development foundation	Type I RIP immunotoxins for killing specific cells	Topic 8/Payload modality
5	No	0	6	US9421256B2	OncXerna therapeutics	Inhibiting VEGF-VEGFR2 interaction for treating cancer and angiogenic diseases	Topic 6/Biological mechanism
6	No	0	5	US20190010229A1	Immuno-Gen	Low-pH surfactant-stabilized formulations for maytansinoid ADCs	Topic 43/Formulation and stability
7	No	0	4	US9198978B2	Immuno-medics	Click-coupled camptothecin conjugation with improved solubility and stability	Topic 6/Payload modality
8	No	0	4	WO2021055306A1	Bristol-myers Squibb	Dual-capture assay for monitoring ADC component transformation	Topic 41/Formulation and stability
9	No	0	4	US12152083B2	Zymeworks	Val-Cit self-immolative linker for adjustable loading across diverse antibody scaffolds	Topic 37/Linker chemistry
10	No	0	4	WO2024081372A1	Gilead Sciences	Process optimization for homogeneous CL2A-SN-38 ADC manufacturing with controlled high DAR	Topic 41/Formulation and stability

Five influential patent concentrate on payload platforms and supporting process or assay infrastructure. US20190010229A1 (Amphlett et al., 2019) discloses a formulation strategy for thiol-containing maytansinoid conjugates such as DM1, based on a low pH succinate buffer with selected surfactants and excipients. The formulation facilitates high concentration liquid formulations and lyophilized products. CN109069665A (Cong et al., 2018) introduces a methylene amino substituent near the maleimide moiety to prevent premature hydrolysis of the distal tubuvaline acetate group. The process enhances plasma stability while preserving lysosomal cleavage of the Val-Cit-maleimide linker, addressing stability limits in tubulysin ADCs. US20230183326A1 (Smith et al., 2023) expands design space through a protein recognition molecule-polymer-drug architecture, in which terminal reactive sites on a polymer scaffold are used to load microtubule inhibitors. This format decouples drug loading from antibody surface hydrophobicity. US7153932B2 (Better et al., 2006) uses type I ribosome-inactivating proteins (RIPs) as toxin modules. The patent describes nucleic acids encoding these proteins, vectors and host cells for constructing immunotoxins by coupling RIPs with antibodies or other cell-binding agents. These immunotoxin systems connect early protein-toxin delivery with ADC design with a broader conjugate platform framework. US12152083B2 (Winters et al., 2024) describes an MT-Val-Cit peptide-based drug-linker toolkit for antimitotic small-molecule toxins bearing aromatic amine self-immolative spacers. It provides a general process for controlled conjugation with 1–8 drug loading sites across diverse antibody scaffolds, including bispecific and anti-HER2 antibodies.

US8034905B2 (Kavlie and Schlunegger, 2011) and US9421256B2 (Kavlie and Schlunegger, 2016) also exhibit high out-degree characteristics and center on humanized antibody compositions that selectively inhibit VEGF-VEGFR2 binding. By inhibiting VEGF binding to VEGFR2, these patents aim to balance efficacy and safety across oncologic and angiogenic disease, such as retinal neovascularization. The patents also describe compositions and administration strategies for expanding indications.

### 3.3 Topic citation landscape

#### 3.3.1 Topic citation network structure

Based on the 47 topics extracted by BERTopic, a directed topic citation network was constructed to analyze structural relationships among ADC technologies. Figure 8 visualizes the integrated network, where each node represents a topic and is labeled by its dominant technological focus (topic label). Directed edges represent topic-level citation relationships aggregated from patent citation data, and edge thickness indicates citation frequency. Node and label sizes were scaled by degree values, so visually prominent nodes correspond to topics with higher citation activity.

**FIGURE 8:**
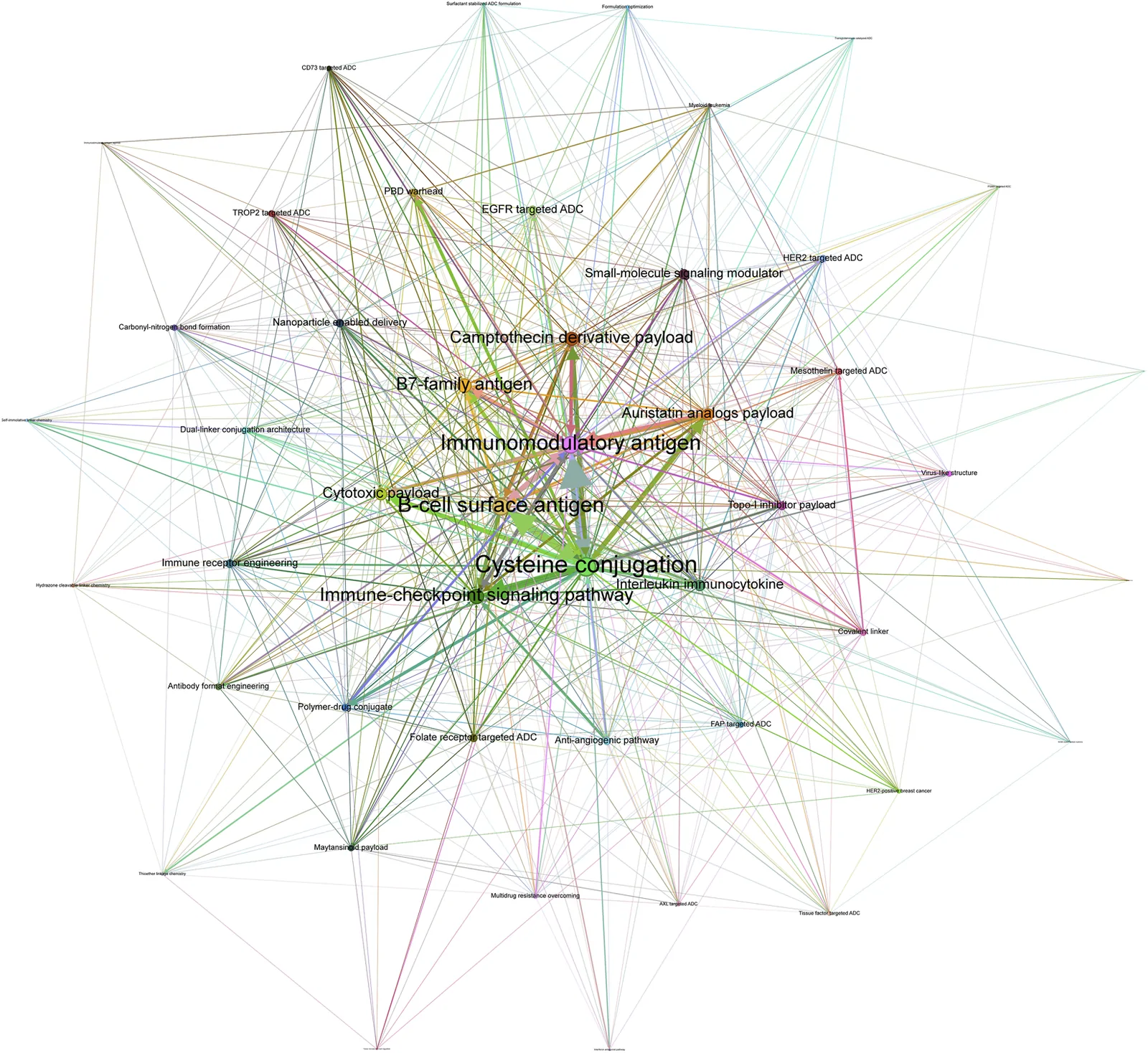
Topic citation network.

The topic citation network displays a core-periphery pattern, with a dense core region and frequent cross references among platform technology groups. Core topics appear repeatedly across downstream invention trajectories, including cysteine-based conjugation (Topic 0), immunomodulatory targets (Topic 1), B-cell surface antigens (Topic 2), immune checkpoint modulation (Topic 3) and B7 family antigens (Topic 4). The core also contains major payload platforms such as camptothecin-derived payloads (Topic 6), auristatin-based payloads (Topic 7), and Topo I inhibitor payloads (Topic 19). These frequently cited topics are located at the center of the network and provides stable technological reference points for subsequent inventions. Target selection, immunobiology, and payload chemistry form the backbone of ADC knowledge.

A ring of medium-sized nodes surrounds the core and covers more specialized target and indication topics. These nodes contain multiple target topics, including EGFR (Topic 11), HER family receptors (Topic 13), TROP-2 (Topic 18), FRα (Topic 21), AXL (Topic 24), FAP (Topic 31), and PSMA (Topic 35). Disease-specific topics in this layer include multiple myeloma (Topic 27), AML (Topic 33), B-cell lymphoma (Topic 40), and HER2-positive breast cancer (Topic 20). These topics retain frequent citation relationships with the core topics, with fewer cross connections among themselves. This pattern indicates that disease and target specific topics repeatedly exploit shared payloads and conjugation strategies, forming application branches on a common technological base.

Periphery nodes are smaller and more sparsely connected, corresponding mainly to specialized chemical strategies and emerging delivery or TME modulation approaches. From a chemical perspective, these nodes cover hydrazone cleavable linkers (Topic 30), thioether linkers (Topic 39), self-immolative linkers (Topic 37), transglutaminase-catalyzed conjugation (Topic 45), and dual-linker systems (Topic 22). Delivery and immunomodulation topics consist of nanoparticle delivery (Topic 16), virus-like carriers (Topic 26), TME modulation (Topic 46), and STING agonist payloads (Topic 42). Despite their overall connectivity is lower, their citation relationships usually connect payload or linker platforms with mechanism or indication clusters. Peripheral technologies support advanced ADC development through knowledge recombination across domains.

The topology of the topic citation network reveals that ADC technological knowledge is organized around a small number of core platform topics. These topics are surrounded by target- and indication-driven application branches, with peripheral chemistry and delivery topics providing further optimization.

#### 3.3.2 Key topics in the network

To identify structurally important topics within the knowledge network, cross-degree and PageRank were calculated for all 47 topics. The cross-degree distribution exhibited a pronounced long-tailed pattern, with a few topics displaying values substantially higher than the remaining topics. Topics with high cross-degree showed strong bidirectional citation activity, reflecting intensive knowledge absorption and extensive downstream dissemination. B-cell surface antigens (Topic 2) and B7 family antigens (Topic 4) illustrate this pattern. Most remaining topics had moderate cross-degree values and occupied localized or peripheral positions in the network.

The 47 topics were ranked by cross-degree, and the top quartile was defined as high cross-degree, yielding 12 candidates. The same top quartile threshold was then applied to PageRank, also yielding 12 topics. The two topic sets partially overlapped and differed in several entries. The intersection contained 10 topics that fell within the top 25% for both cross-degree and PageRank (Table 4). These topics were defined as key topics for subsequent analysis. The 10 key topics included five target-related topics: Topic 2, B-cell surface antigens; Topic 4, B7 family antigens; Topic 15, Mesothelin; Topic 24, AXL; and Topic 13, HER family receptors). The remaining topics covered two payload platforms: Topic 9, PBD payloads, and Topic 6, Camptothecin-derived payloads; two immune mechanism topics: Topic 5, IL immunocytokine, and Topic 3, Immune checkpoint modulation; and one delivery strategy topic: Topic 16, Nanoparticle delivery.

**TABLE 4:** Key topics in ADC patent landscape.

Topic ID	Topic label	Cluster	Cross-degree (λ = 0.5)	Page rank	In-degree	Out-degree	r_diff_	Role
2	B-cell surface antigens	Target selection	318.66	0.105	259	392	0.398	Absorption-oriented
4	B7 family antigens	Target selection	238.60	0.110	298	191	0.609	Diffusion-oriented
9	PBD payloads	Payload modality	203.18	0.074	192	215	0.472	Bridging
6	Camptothecin-derived payloads	Payload modality	135.71	0.064	177	104	0.630	Diffusion-oriented
5	Interleukin immunocytokines	Biological mechanism	110.78	0.047	118	104	0.532	Bridging
3	Immune checkpoint modulation	Biological mechanism	101.86	0.034	91	114	0.444	Absorption-oriented
15	Mesothelin	Target selection	100.19	0.032	79	127	0.383	Absorption-oriented
24	AXL	Target selection	72.84	0.045	115	46	0.714	Diffusion-oriented
13	HER family receptors	Target selection	71.42	0.039	91	56	0.619	Diffusion-oriented
16	Nanoparticle delivery	Delivery system	48.30	0.021	44	53	0.454	Bridging

* Underlined values indicate topics with r_diff_ below the threshold.

Beyond the 10 key topics, four additional topics demonstrated single-metric structural significance. Two topics had high cross-degree but were below the PageRank threshold: Topic 34, Carbonyl-amine coupling, and Topic 18, TROP2. The other two topics had high PageRank values: Topic 25, Multidrug resistance, and Topic 11, EGFR. Since these topics did not rank highly on both metrics, their structural importance in the overall network was more localized than that of the key topics.

#### 3.3.3 Topic roles in knowledge flow

A comparison of in-degree and out-degree within the topic citation network clarified the participation of structurally important topics in knowledge flows. Building on the diffusion ratio $r_{diff}$ , the identified key topics were assigned to three knowledge flow roles. Topics with $r_{diff}\geq 0.55$ were regarded as diffusion-oriented, and those with $r_{diff}\leq 0.45$ were designated as absorption-oriented. Topics with ${0.45\;<\;r}_{diff}\;<\;0.55$ and both in-degree and out-degree above the network median were considered bridging topics.

Diffusion-oriented topics had higher in-degree than out-degree, indicating that their knowledge was repeatedly reorganized by subsequent inventions. Among the key topics, this group contained B7 family antigens (Topic 4, in-degree = 298, out-degree = 191), camptothecin-derived payloads (Topic 6, in-degree = 177, out-degree = 104), AXL (Topic 24, in-degree = 115, out-degree = 46), HER family receptors (Topic 13, in-degree = 91, out-degree = 56). These topics represented relatively mature design platforms and therapeutic principles, including B7 family antigens, HER/EGFR/AXL-related signaling, and camptothecin-derived Topo I inhibitor payloads. They provided stable reference points for subsequent inventions. A similar diffusion profile was observed for additional structural significance topics, such as EGFR (Topic 11) and multidrug resistance (Topic 25). Both achieved high PageRank scores alongside incoming citation flows that exceeded outgoing connections. Within the topic citation network, diffusion-oriented topics represented major diffusion centers that supported downstream reorganized of established knowledge.

Absorption-oriented topics displayed higher out-degree than in-degree, reflecting intensive citation and integration of upstream knowledge relative to their citation impact. Within the key topic set, B-cell surface antigens (Topic 2, in-degree = 259, out-degree = 392), immune checkpoint modulation (Topic 3, in-degree = 91, out-degree = 114), and mesothelin (Topic 15, in-degree = 79, out-degree = 127) illustrated this behavior. Their high out-degree and topological positions at the intersections of multiple topics indicated reliance on a broad range of upstream topics. In ADC evolution, topics centered on targets and mechanisms represented knowledge absorbers, connecting different strands of upstream research. Similar absorption-oriented patterns appeared in additional topics, including TROP-2 (Topic 18, in-degree = 33, out-degree = 63) and carbonyl-amine coupling (Topic 34, in-degree = 40, out-degree = 60).

A third category bridged different parts of the knowledge space. Bridging topics had relatively balanced in-degree and out-degree, and also achieved high cross-degree and PageRank scores. Bridging topics occupied interface positions in the topic citation network, where multiple knowledge streams intersected. PBD payloads (Topic 9, in-degree = 192, out-degree = 215), IL immunocytokine (Topic 5, in-degree = 118, out-degree = 104), and nanoparticle delivery (Topic 16, in-degree = 44, out-degree = 53) were representative examples. The payload platforms, immunomodulators, and delivery systems represented by these topics were universally adaptable and participated in the upstream and downstream knowledge. PBD and other DNA-damaging payload platforms connected diverse antigens and indications. IL-based immunocytokines bridged targeted cytotoxicity with systemic immunomodulation. Nanoparticle delivery systems connected chemically defined ADC architectures with nanomedicine and drug delivery technologies. These topics did not necessarily have the highest citation counts. Their interface positions and high cross-degree allowed knowledge exchange between weakly coupled regions of the topic space, facilitating cross-domain recombination and new ADC design paradigms.

## 4 Discussion

The key topics identified in the topic citation network are hubs because their structural centrality reflects sustained knowledge circulation, repeated technical integration, and broad connections across development trajectories. Their central positions indicate that they concentrate recurring design principles, engineering and translational priorities that have shaped ADC development. These recurring patterns span target selection, payload construction, immune regulation, and delivery engineering. A detailed examination of these hubs helps clarify the current state of technology development, clinical translation, and pipeline expansion. A hub perspective highlights the engineering principles that are recombined and refined within the evolving ADC technological trajectory.

### 4.1 Target-centered hubs

The five target-centered hubs identified in this study were B-cell surface antigens (Topic 2), B7 family antigens (Topic 4), mesothelin (Topic 15), AXL (Topic 24), and HER family receptors (Topic 13). Collectively, these antigen platforms anchor main areas of current ADC development. These hubs closely mirror contemporary drug development, where major pharmaceutical companies extend validated antigen platforms with remaining clinical and commercial space. Development therefore concentrates on biological characterization, indication selection, and product differentiation around these antigen platforms.

From a technological perspective, B-cell and B7 family hubs capture ADC targeting in hematologic malignancies and immune regulatory pathways. B-cell associated antigens such as CD19, CD20, and CD22 are classical B-cell markers. Their expression patterns, internalization profiles, and ADC suitability differ across targets, making target specific evaluation necessary. Several B-cell ADCs have been approved, including loncastuximab tesirine (CD19), inotuzumab ozogamicin (CD22), and polatuzumab vedotin (CD79b). These agents direct potent cytotoxic payloads to defined B-cell markers and support the clinical value of B-cell target platforms in lymphoma and related diseases (Liu et al., 2024). Developed by companies such as Pfizer, Roche/Genentech, and ADC Therapeutics, these products established reusable design principles based on stable expression, adequate internalization, and compatibility with multiple linker-payload systems.

The B7 family hub covers co-stimulatory and co-inhibitory immune checkpoint ligands, represented by B7-H3 and B7-H4, which are overexpressed in many tumors and associated with immune escape. In recent years, B7-H3 ADC pipelines have expanded rapidly. Representative programs include ifinatamab deruxtecan, co-developed by Daiichi Sankyo and Merck, and HS-20093/GSK5764227, developed through the Hansoh-GSK collaboration. These programs place B7 family antigens in small-cell lung cancer, neuroendocrine tumors, and other solid malignancies (Qiu et al., 2021).

The HER2, mesothelin, and AXL hubs illustrate target platform consolidation and product differentiation in solid tumors. HER2 is an established oncogenic receptor and a central ADC platform, with multiple payloads, linkers, and dosing regimens developed around this antigen backbone. HER2 remains one of the most commercially successful ADC targets. Several approved HER2-directed ADCs include T-DM1, T-DXd, disitamab vedotin, and trastuzumab rezetecan (SHR-A1811), trastuzumab botidotin (A166). Other HER2 ADCs, such as trastuzumab duocarmazine (SYD985), reached late-stage development or regulatory review, although its EMA marketing authorization application was withdrawn. Among them, T-DXd, commercialized as Enhertu by AstraZeneca and Daiichi Sankyo, has reshaped treatment paradigm in HER2-positive and HER2-low breast cancer, and has expanded clinical use across gastric cancer, lung cancer, and other solid tumors (Abelman et al., 2022). Its global sales and multiple Phase III trials have intensified competition around the HER2 ADC platform and accelerate optimization of payload selection, linker chemistry, and dosing strategies.

Mesothelin and AXL represent targets for invasive and biologically complex solid tumors. Mesothelin is strongly expressed in mesothelioma, ovarian cancer, and several other solid tumors, with limited expression in normal tissues. Multiple mesothelin ADCs have entered clinical development, including anetumab ravtansine, BMS-986148, HBM9033, and RC88. Early studies have shown manageable safety and modest or preliminary efficacy. The tolerability and encouraging activity reported for RC88 reinforce the suitability of mesothelin as an ADC target in solid tumors. The AXL ADC mecbotamab vedotin (BA3011) uses a conditionally active biologic format designed to reduce off-target toxicity (Yadav et al., 2025). BA3011 has been entered Phase II trials in refractory solid tumors that progressed after PD-1/PD-L1, EGFR-TKIs, or ALK inhibitors. AXL is associated with resistance to immune checkpoint inhibitors (ICIs). Synergistic effects between AXL ADCs and immunotherapy are being explored, as AXL inhibition may reverse immunosuppressive signaling in the TME and enhance ICI sensitivity (Zheng et al., 2025). These findings support further evaluation of combination therapy.

Taken together, these five target-centered hubs indicate that ADC innovation currently concentrates on recombination and extension around validated antigen platforms. These platforms attract substantial research resources and commercial investment. Major pharmaceutical companies, represented by Roche/Genentech, AstraZeneca, Daiichi Sankyo, Pfizer, GSK, and Bristol Myers Squibb, occupy dominant positions in this field. Emerging regional biotechnology companies, particularly in China, are rapidly advancing ADC pipelines targeting HER2 and B7 family antigens. Differentiation in these competitive markets is increasingly pursued through payload selection, linker technologies, combination regimens, and treatment-line positioning within targets hubs.

Resistance at the target recognition stage may arise from reduced or heterogeneous antigen expression, antigen loss, or insufficient internalization, which limits ADC binding and intracellular payload delivery (Loganzo et al., 2016; Li et al., 2025). Bispecific and multispecific ADCs provide a next-generation strategy for addressing some of these limitations. Biparatopic ADCs recognize two epitopes on the same antigen and may promote receptor aggregation and internalization, whereas dual-antigen ADCs can expand the coverage of tumor cells when antigen expression is heterogeneous (Gu et al., 2024). These designs are particularly relevant to established solid tumor targeting platforms, including HER2, B7 family antigens, mesothelin, and AXL. Nevertheless, their therapeutic efficacy depends on the synergistic optimization of antigen combination, binding geometry, internalization behavior, antibody format, and linker-payload properties. Bispecific and multispecific architectures should therefore be viewed as extensions of validated targeting platforms.

The industrial configuration delineates specific strategic trajectories for future research. Novel ADC targets are more likely to receive sustained attention and resources when their biology can be connected with validated antigen platforms, including B-cell antigens, B7 family, HER2, mesothelin, and AXL. Systematic characterization of target expression heterogeneity, endocytosis dynamics, and resistance mechanisms will be critical for selecting the next-generation targets and designing indication strategies. Coupling target hubs with non-target technological domains will shape the clinical positioning and corporate pipelines of novel ADC therapies. Multi-mechanism ADC platforms may be developed through integration of target selection, payload design, immune modulation, and delivery strategies.

### 4.2 Payload chemistry hubs

The two payload chemistry hubs identified in this study were PBD payloads (Topic 9) and camptothecin-derived payloads (Topic 6). These topics represent central cytotoxic platforms that shape payload design. In the topic citation network, both hubs exhibited high cross-degree and PageRank scores, highlighting central positions in payload-related knowledge diffusion. PBDs and camptothecin derivatives can be paired with diverse antibody-target configurations as cytotoxic modules and chemical backbones. Linker engineering, conjugation strategies, and dosing regimens built on these backbones have become major drivers of ADC technological evolution.

PBDs, particularly PBD dimers, are among the most representative ultrapotent DNA-interacting payloads. PBD dimers act through DNA minor-groove binding and interstrand crosslinking, a mechanism distinct from microtubule inhibitors and DNA cleavage. Loncastuximab tesirine, marketed as Zynlonta by ADC Therapeutics, is a CD19-directed PBD-based ADC. LOTIS-2 study reported clinically meaningful responses in patients with relapsed or refractory DLBCL after multiple prior therapies. These results validate the potential of PBD payloads to induce deep tumor debulking in highly refractory populations. The extreme potency of PBD-based ADCs constrains clinical dosing and narrows therapeutic windows. Common toxicities include myelosuppression, liver function abnormalities, edema, and cutaneous reactions. Adverse events have led to discontinuation or dose-limiting decisions in certain pipelines. The primary challenge for next-generation engineering is balancing extreme potency against limited tolerability. Current strategies involve reduced-potency PBD dimers and prodrug concept (proPBD ADCs). Advanced proPBD designs shields the PBD payload with capping groups that cleave preferentially in the TME (Liu B. et al., 2025b).

Camptothecin derivatives provide a rapidly expanding Topo I payload platform with a mechanism distinct from PBDs. Common camptothecin-derived ADC payloads include SN-38 and exatecan derivatives (e.g., DXd). Related products, particularly SG and T-DXd, have shown substantial clinical activity in solid tumors, including breast, gastric, and lung cancers. Clinical success has positioned Topo I inhibitor payloads as cross-target platforms with pan-tumor applicability. Compared with DNA-alkylating agents, camptothecin-based payloads can offer a broader therapeutic window. Topo I ADCs combine moderately potent, membrane-permeable payloads with tumor-selective cleavable linkers and relatively high DAR values (Goundry et al., 2024), supporting the bystander effect and tumor penetration for sustained efficacy. Topo I ADCs are associated with adverse events, such as neutropenia, diarrhea, and interstitial lung disease (particularly T-DXd). Free payloads exposure in plasma and tissue interstices may contributed to these events. Stable hydrophilic linkers and conjugation strategies can help balance efficacy and tolerability. The growing Topo I ADC pipeline confirms that payload diversification has become a major direction in current ADC development.

Camptothecin derivatives provide a rapidly evolving Topo I payload platform with development profile distinct from that of PBDs. The ultrahigh potency and long-lasting DNA crosslinking produced by PBD dimers supports tumor cell killing at low intracellular concentrations, but also leave limited tolerance to premature payload release or nonspecific tissue exposure. This narrow therapeutic window constraints the combinations of targets, linkers, DAR, and dosing regimen that can be safely developed (Hartley, 2021). In contrast, the more moderate intrinsic potency of camptothecin-derived payloads provides greater flexibility for relatively high DAR and clinically manageable dosing. Derivatives such as SN-38 and the exatecan-based DXd can also be incorporated into cleavable linker systems that release membrane permeability payloads. The released payload can diffuse to neighboring cells, allowing bystander effect to partially compensate for heterogeneous or low antigen expression in solid tumors (Khera et al., 2022; Pommier and Thomas, 2023). Moreover, camptothecin scaffolds can be chemically modified to modulate potency, solubility, stability, and membrane permeability, facilitating their integration with different antibodies and linkers. These properties help explain the wide applicability of camptothecin-derived payloads across different target platforms and solid tumor indications, as well as in subsequent ADC development. This broader technological applicability should not, however, be interpreted as evidence of inherently superior clinical efficacy.

Payload-related resistance may involve increased drug efflux, altered payload targets, enhanced DNAdamage repair, or reduced susceptibility to payload-induced cell death (Loganzo et al., 2016; Li et al., 2025). Dual- and multi-payload ADCs attempt to address this problem by delivering two agents with distinct pharmacological properties or mechanisms by the same antibody. In a preclinical study, homogeneous HER2-targeted ADCs carrying MMAE and MMAF produced better tumor control and survival benefits than the corresponding single-payload ADCs or their combination in a xenograft model with intratumoral HER2 heterogeneity and resistance (Yamazaki et al., 2021). Although both payloads inhibit microtubules, their different membrane permeability and intracellular retention suggest that the dual payload design can combine complementary pharmacological properties. Translation of this approach nevertheless requires control of coupling homogeneity, total and payloadspecific DAR, hydrophobicity, release kinetics, and potential overlapping toxicity (Tao et al., 2025).

Resistance may also develop downstream of payload delivery through alterations in tumor-cell death pathways. In HER2-low breast cancer models, crVDAC3 bound to HSPB1 and inhibited its ubiquitin-mediated degradation, leading to HSPB1 accumulation, suppression of ferroptosis, and resistance to T-DXd. Disruption of crVDAC3-HSPB1 interaction by paritaprevir promoted HSPB1 degradation and enhanced T-DXd activity in patient-derived xenograft models (Zou et al., 2024). This study suggests that overcoming ADC resistance may require intervention in the cellular response to payload-induced damage. However, the clinical relevance and its generalizability of this mechanism across ADC platforms remain to be determined.

The payload chemistry hubs identified in the topic citation network indicate that future ADC technology will not rely solely on potent cytotoxic agents. Progress depends on rational integration of target biology, payload physicochemical properties, linker structures, and delivery strategies. For PBD payloads, successful clinical translation hinges on maintaining antitumor activity in refractory disease while controlling systemic toxicity through dose optimization and conjugation chemistry. For camptothecin derivatives, the priority is to minimize nontumor tissue exposure, address mechanism-specific adverse events, and clarify target-payload settings in which bystander effect provides clear therapeutic advantages (Khera et al., 2022). High-frequency knowledge exchanges around these payload hubs and their peripheral topics may drive differentiated pipeline strategies. Such interaction may propel multi-mechanism platform that combine cytotoxicity, the bystander effect, immune regulation, and delivery engineering.

### 4.3 Immune mechanism hubs

The two immune mechanism hubs identified in this study, immune checkpoint modulation (Topic 3) and IL immunocytokines (Topic 5), highlight the close coupling among ADC technologies, cancer immunotherapy, and TME modulation. Within the ADC technology system, these hubs define immune interfaces. They surpass conventional antibody-payload modules by connecting targeted cytotoxic effects with host immune responses at the TME level.

Immune checkpoint pathways such as PD-1/PD-L1 and CTLA-4 regulate T-cell activity. Corresponding ICIs have produced durable responses in melanoma, lung cancer, urothelial carcinoma, and selected breast cancer settings. Mechanistic studies support the rationale for combining ADCs with ICIs, because ADC payloads can trigger immunogenic cell death (ICD) and activate immune responses. After exposure to specific cytotoxic payloads, dying tumor cells release TAAs and damage-associated molecular patterns (DAMPs). DAMPs include surface-exposed calreticulin, heat shock proteins, extracellular ATP, HMGB1, and proinflammatory cytokines (Lv et al., 2025). These signals promote phagocytic uptake of dying cells, enhance antigen presentation, increase CD8^+^ T-cell infiltration, and may upregulate PD-L1 expression. The TME may therefore become more responsive to checkpoint blockade. Multiple clinical trials combining SG or T-DXd with pembrolizumab or other PD-1/PD-L1 inhibitors have reported encouraging objective response and disease control outcomes in selected previously treated or PD-L1-positive populations.

Adaptive signaling and phenotypic plasticity may also reduce ADC sensitivity after adequate target binding and payload delivery. In hormone receptor-positive breast cancer models, MMP1 activated NF-κB signaling, promoted epithelial-mesenchymal transition, and contributed to resistance to SG. Genetic suppression of MMP1 restored SG sensitivity in resistant cellular and xenograft models (Chen et al., 2025). These findings identify the MMP1-NF-κB-EMT axis as a potential resistance mechanism, although its predictive and therapeutic relevance in patients requires further validation. A distinct relationship between ADC and treatment resistance was demonstrated for RC48 in bladder cancer. This study demonstrates that RC48 could confront an HER2-associated immunosuppressive microenvironment and enhance responsiveness to immune checkpoint blockade. RC48 reduced PD-L1 transcription through Hippo-TAZ signaling, promoted the release of CCL5, CXCL9, and CXCL14, recruits cytotoxic T cells, and enhanced the activity of CTLA-4 and PD-L1 antibodies in preclinical models (Xiao et al., 2025). Thus, immune-related ADC strategies may target adaptive processes that reduce ADC sensitivity or exploit ADC-mediated remodeling of the TME to overcome resistance to other treatment modalities.

Since standard combination therapies may augment immune-related adverse events (irAEs), immune-modulating ADCs (IDCs) have emerged as an alternative treatment format. Unlike conventional designs that rely primarily on overexpressed tumor antigens, IDCs directly recognize immune escape molecules such as PD-L1, B7-H3, and B7-H4. IDCs aim to partially reverse local immune suppression while delivering cytotoxic payloads to tumor cells and immunosuppressive TME components, generating synergistic antitumor effects (He et al., 2021). Several IDCs have shown preclinical or early activity. PDL1V(PF-08046054), the first PD-L1 ADC, has entered Phase III trials, whereas domestically developed HLX43 has advanced into Phase II and Phase II/III clinical studies. Experimental data suggest that effector T cells can tolerate certain small-molecule payloads within appropriate dose ranges through multidrug resistance transporters. Macrophages and dendritic cells may require intracellular concentrations approximately 100-fold higher to undergo lethal damage, which may help keep systemic toxicity of PD-L1 ADCs manageable (Gay et al., 2024). Immune checkpoint molecules represent by PD-1, CD70, and CD73 are also expressed on normal immune cells. On-target/off-tumor toxicity may thus lead to irAEs, hematologic toxicity, and organ damage, requiring careful consideration.

Interleukin immunocytokines embody another coupling strategy centered on cytokines and their receptors. This hub includes conjugates directed at cytokine receptor biology and antibody-cytokine or antibody-linker-payload designs for localize immune activation. Localized delivery can remodel pathological immune circuits while limiting systemic exposure. In oncology, ADCs targeting CD25 or CD123 have been developed to selectively eliminate regulatory T cells (Tregs) or abnormal hematopoietic stem/progenitor cells within disease-associated microenvironments (Zammarchi et al., 2020). The goal is to enhance effector T-cell responses and restore local immune balance. Antibody-cytokine fusion proteins (IL-2- or IL-12-based immunocytokines) can amplify local immune activation and improve safety margins through tumor site accumulation.

Beyond oncology, immune-modulating ADCs have been extended to autoimmune and inflammatory diseases. For instance, blood dendritic cell antigen 2 (BDCA2) ADCs can selectively deliver glucocorticoid receptor modulators (GRMs) to plasmacytoid dendritic cells. Candidate agents like DB-2304 inhibited type I interferon signaling and inflammatory cytokine release *in vitro* and in preclinical models, and downregulated interferon response gene signatures in systemic lupus erythematosus models. This approach may retain immunosuppressive activity while reducing systemic glucocorticoid exposure (Li et al., 2024). Autoimmune ADCs targeting IL-4R primarily address diseases dominated by T helper 2 (Th2) inflammation. IL-4R is expressed on multiple immune cells, including B cells, Th2 cells, mast cells, eosinophils, as well as structural cells (e.g., fibroblasts), and plays a central role in type II inflammatory diseases (Dixit et al., 2024). Recent patents and preclinical studies have explored IL-4R antibodies conjugated to glucocorticoid analogues or GRMs to confine immunosuppression to sites with hyperactivation of the IL-4/IL-13 axis. Local payload delivery may maintain therapeutic efficacy while reducing systemic risks associated with long-term steroid therapy. Immunology ADCs targeting TNF-α, including ABBV-3373 and ABBV-154, use adalimumab conjugated to a GRM payload. These agents were designed to direct glucocorticoid activity to activated immune cells and showed disease control and broader cytokine suppression than anti-TNF monotherapy in rheumatoid arthritis and polymyalgia rheumatica.

Subsequent studies raised concerns about biomarker alterations and infection risk, consistent with possible systemic glucocorticoid exposure at higher doses or longer durations. The benefit-to-risk ratio failed to show sufficient differentiation from existing IL-6 or JAK inhibitors, leading to the discontinuation of ABBV-154. Taken together, these experiences underscore that autoimmune ADCs require a clinically verifiable balance between local efficacy at lesions and long-term systemic safety.

The immune mechanism hubs identified in this study broaden the criteria for evaluating ADC success. In cancer treatment, tumor shrinkage and objective response rates should be evaluated alongside response durability and immune activation. Durable antitumor immunity is becoming an important evaluation dimension for immune-related ADCs. For autoimmune and inflammatory diseases, extensive systemic immunosuppression is gradually being replaced by cell subset selectivity and localized immune intervention. Advances in immune checkpoint modulation and interleukin immunocytokines suggest that future ADC design will prioritize balanced optimization of target biology, payload properties, linker architecture, delivery routes, and immune regulatory mechanisms. Such optimization may support more integrated therapeutic engineering of ADCs and immunomodulatory conjugates.

### 4.4 Delivery strategy hub

Nanoparticle delivery (Topic 16) illustrates the integration of ADC design with pharmacokinetic optimization, tissue distribution, and tumor penetration. Patents in this field revolve around nanoparticles-based engineering solutions that increase lesion-site exposure and reduce systemic toxicity. Several nanoplatforms are under active investigation, including liposomes, polymeric micelles, inorganic nanoparticles, and albumin-based nanocarriers.

Antibody-conjugated nanoparticles are formed by attaching antibodies to the nanoparticle surface. They combine high drug loading capacity of nanocarriers with the antibody-mediated molecular specificity. The CD20-targeted AR160 combines nab-paclitaxel nanoparticles with rituximab in a nano-immunoconjugate. Preclinical models showed superior antitumor activity and tolerability compared with conventional nab-paclitaxel. These findings validate the potential of ADC-nanoparticle hybrid architectures to enhance tumor enrichment, prolong circulation, and enable stimulus-responsive release (Habermann et al., 2023). Research on antibody-drug nanoparticles targeting HER2-positive breast cancer further demonstrates the integration of antibodies and multidrug modules within the same nanostructure. The integrated design may improve pharmacokinetic and tissue distribution profiles while maintaining or enhancing antitumor effects.

Albumin-based nanoparticles can modify the therapeutic profile of cytotoxic agents through delivery engineering. Albumin possesses high abundance, a long half-life, and good biocompatibility. Albumin can undergo gp60/caveolae-mediated endothelial transcytosis and interact with secreted protein acidic and rich in cysteine (SPARC) in the tumor stroma, supporting preferential tumor accumulation. This mechanism improves paclitaxel distribution *in vivo* and provides a more controllable safety window than solvent-based paclitaxel. Clinical application of albumin-bound paclitaxel in several cancers has supported the transformative value of albumin-based delivery. Related research has advanced through clinical evaluation, mechanism validation, and next-generation albumin delivery systems involving gp60/SPARC pathways.

In tumor immunotherapy, nanodelivery strategies increasingly intersect with immune modulation. Numerous preclinical studies have explored co-encapsulation of chemotherapeutics with TLR or STING agonists in liposomal or polymeric nanocarriers. Examples include degradable nanoparticles or liposomes co-loaded with doxorubicin and TLR agonists such as poly(I:C) or R848. Co-delivery can induce ICD, enhance antigen presentation, and strengthen innate immune activation and type I interferon signaling at the tumor site. These systems integrate chemotherapy and immunotherapy within a single co-delivery platform. Research on STING agonist nanodelivery indicates that nanoparticle formulations can improve membrane permeability, plasma stability, and tumor targeting of STING agonists. These formulations may reduce systemic toxicity and enhance antitumor activity when combined with PD-1/PD-L1 inhibitors in solid tumor models.

Delivery-related resistance may occur even when antigen expression and intrinsic payload sensitivity are retained. Inefficient internalization, recycling or altered endosomal trafficking of ADC–antigen complexes, impaired lysosomal acidification or proteolysis, and defective intracellular payload release can all reduce the amount of active drug reaching the site of action (Loganzo et al., 2016; Li et al., 2025). These limitations provide a rationale for delivery systems that rely less on a single internalization and lysosomal processing pathway. TME-responsive linker allows extracellular release in response to tumor-associated enzymes or other local conditions, whereas noninternalized ADCs are designed to release membrane-permeable payloads near poorly internalizing antigens or extracellular tumor components (Ashman et al., 2022). Nanoparticle and carrier forms may provide additional control over tissue distribution and payload release. However, these methods must maintain sufficient plasma stability and tumor selectivity to avoid premature or off-tumor payload exposure.

From a perspective of technological evolution and pipeline strategy, next-generation ADCs and ADC-related conjugates can be viewed as components of larger delivery systems. Two major challenges in nanoparticle delivery are balancing prolonged circulation with tumor penetration and aligning payload mechanisms with pH-, enzyme-, or other stimulus-responsive release systems. As nanoparticle delivery converges with other core technologies, delivery engineering is likely to become central to efficacy improvement, indication expansion, and diversified ADC pipeline strategies.

Several methodological factors should be considered when interpreting these findings. BERTopic was applied to patent titles and abstracts that capture the principal technical disclosures but do not contain all details presented in claims or specifications. Topic granularity is influenced by the embedding model, dimensionality reduction and clustering parameters, domain-informed seed topics, automatic topic reduction, and subsequent topic label interpretation. Semantically ambiguous or low-frequency inventions may be classified as noise or incorporated into broader topics, potentially reducing the visibility of emerging technologies with a limited number of patents. In addition, patent citation patterns are affected by review practices, citation age, and differences among patent authorities. Therefore, topic size, network centrality, and knowledge-flow roles are not direct measures of technical quality, commercial value, or clinical efficacy. Future studies incorporating full patent texts, multilingual semantic models, and multi-label topic assignments may provide a more detailed representation of the ADC technology landscape.

## 5 Conclusion

This study mapped the ADC technological landscape through an integrated framework combining BERTopic with patent citation network analysis. A total of 47 topics were identified and further organized into nine technological clusters, outlining a structured innovation system across target biology, antibody architecture, biological mechanisms, conjugation chemistry, payload modality, delivery systems, and formulation strategies. The patent and topic citation networks identified a limited set of structurally important patents and hub topics. These hubs were concentrated around major targets, payload platforms, immune mechanisms, and nanoparticle delivery strategies. They exhibited high connectivity across technological domains and organize knowledge reuse, integration, and trans-topic reorganization across the ADC innovation ecosystem. Overall, this study provides an evidence-based view of the ADC patent landscape and a structured perspective on its technological trajectory. The proposed framework also offers a replicable approach for identifying key technologies and supporting strategic decision-making in target prioritization, payload design, and R&D planning.

## Data Availability

The original contributions presented in the study are included in the article/Supplementary Material; further inquiries can be directed to the corresponding authors.
